# Visible-light promoted cascade annulation of *N*-propargylamines with sodium sulfinates to access sulfonylated 9*H*-pyrrolo[1,2-*a*]indoles and quinolines

**DOI:** 10.1039/d6ra01687a

**Published:** 2026-04-29

**Authors:** Junke Wu, Hongqiang Dong, Zhuo Li

**Affiliations:** a College of Agriculture, Tarim University Alaer 843300 China donghongqiang@taru.edu.cn; b Shandong Key Laboratory for Green Prevention and Control of Agricultural Pests, Institute of Plant Protection, Shandong Academy of Agricultural Sciences Jinan 250100 China lizhuo0613@163.com

## Abstract

A photo-initiated efficient protocol for the synthesis of sulfonylated 9*H*-pyrrolo[1,2-*a*]indoles and quinolines from *N*-propargylamines and easily available sodium sulfinates has been reported. This approach uses mild reaction conditions, requires no transition metal catalysis, photocatalyst, and shows a broad substrate scope.

## Abstract 2

A photo-initiated efficient protocol for the synthesis of sulfonylated 9*H*-pyrrolo[1,2-*a*]indoles and quinolines from *N*-propargylamines and easily available sodium sulfinates has been reported.

## Graphical abstract

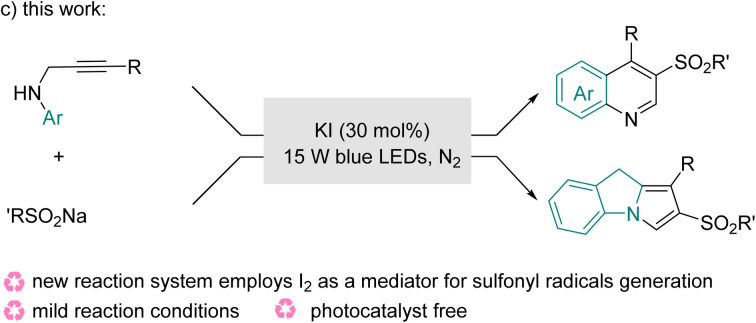

Two centuries ago, during the early development of organic chemistry, the significance of heterocycles in living systems was already acknowledged, exemplified by the isolation of alkaloids like morphine from poppy seeds, quinine from cinchona bark, and camptothecin from the Camptotheca tree. A 2024 review by Njardarson *et al.* of U.S. FDA-approved heterocycle-containing drugs (2013–2023) found that 82% of small-molecule drugs incorporated at least one heterocycle, marking a substantial rise from the previous level of 59%.^1^ Among them, indole, pyrrolidine and quinoline are some of the most frequently occurring nitrogen-containing heterocycles. Therefore, the synthesis and modification of nitrogen-containing heterocycles have become a continuously popular research topic.^2^ Undoubtedly, visible-light-driven organic reactions have emerged as one of the most vibrant research areas over the past two decades.^3,4^ Characterized by mild conditions, high efficiency, and broad compatibility, such reactions offer a powerful and versatile platform for modern synthesis. Very recently, radical cascade cyclization of C–C double and triple bonds has significantly advanced synthetic methodologies for polycyclic nitrogen heterocycles.^5^ For example, Yang and Meng developed a visible-light-induced EDA-complex enabled radical cascade perfluoroalkylation/cyclization reaction for the synthesis of perfluoroalkyl-substituted pyrrolidines from *N*-cyanamide alkenes and perfluoroalkyl iodides.^6^ In 2025, Yu and Pan reported another photoinduced 4CzIPN-catalyzed cascade difluoroalkylation/cyclization of *N*-allylamines, which enables the construction of C2-difluoroalkylated pyrroloquinazolinones.^7^ In addition, a silver-catalyzed oxidative cyclization of 1,6-enynes and sodium sulfinates has been established by Wu and Jiang's group to access sulfonylated benzofurans.^8^ However, current synthetic routes suffer from several drawbacks, including the need for transition metal catalysts, harsh reaction conditions, and challenging post-processing requirements.

On the other hand, organosulfone derivatives are widely distributed in a diverse range of natural products, bioactive molecules, and functional materials.^9^ Due to their proven value in pharmaceuticals and agricultural chemicals, developing efficient synthetic routes to sulfone-containing compounds remains an area of significant interest. Recently, the sulfonyl radical-initiated cascade reactions of alkenes and alkynes for the construction of sulfone-containing compounds have emerged as a prominent area of research.^10^ Generally, electrochemistry, photochemistry, or oxidants trigger the *in situ* generation of sulfonyl radicals in the above transformations. In addition, *N*-propargylamines have proven to be excellent substrates in radical cascade reactions, and significant synthetic advances over the past decade have enabled efficient access to diverse molecular skeletons.^11^ For example, Chen and Li reported a photo-promoted EDA complex enabled oxidative cyclization of *N*-propargylanilines with sulfinic acids to obtain 3-sulfonated quinoline derivatives.^11*g*^ Recently, Yan, Sun, Li, and colleagues disclosed another photo-induced radical silylation cyclization of *N*-propargylindoles with silylboronates or tris(trimethylsilyl)silane, to give silylated fused cycles.^11*n*^ However, to the best of our knowledge, approaches for the preparation of sulfonylated polycyclic nitrogen heterocycles from *N*-propargylamines and easily available sodium sulfinates has less been reported. In this work, we herein develop a photocatalyst-free, efficient and eco-friendly strategy to gain sulfonated 9*H*-pyrrolo[1,2-*a*]indoles and quinoline derivatives from *N*-propargylamines and sodium sulfinates (Scheme 1c).

**Scheme 1:**
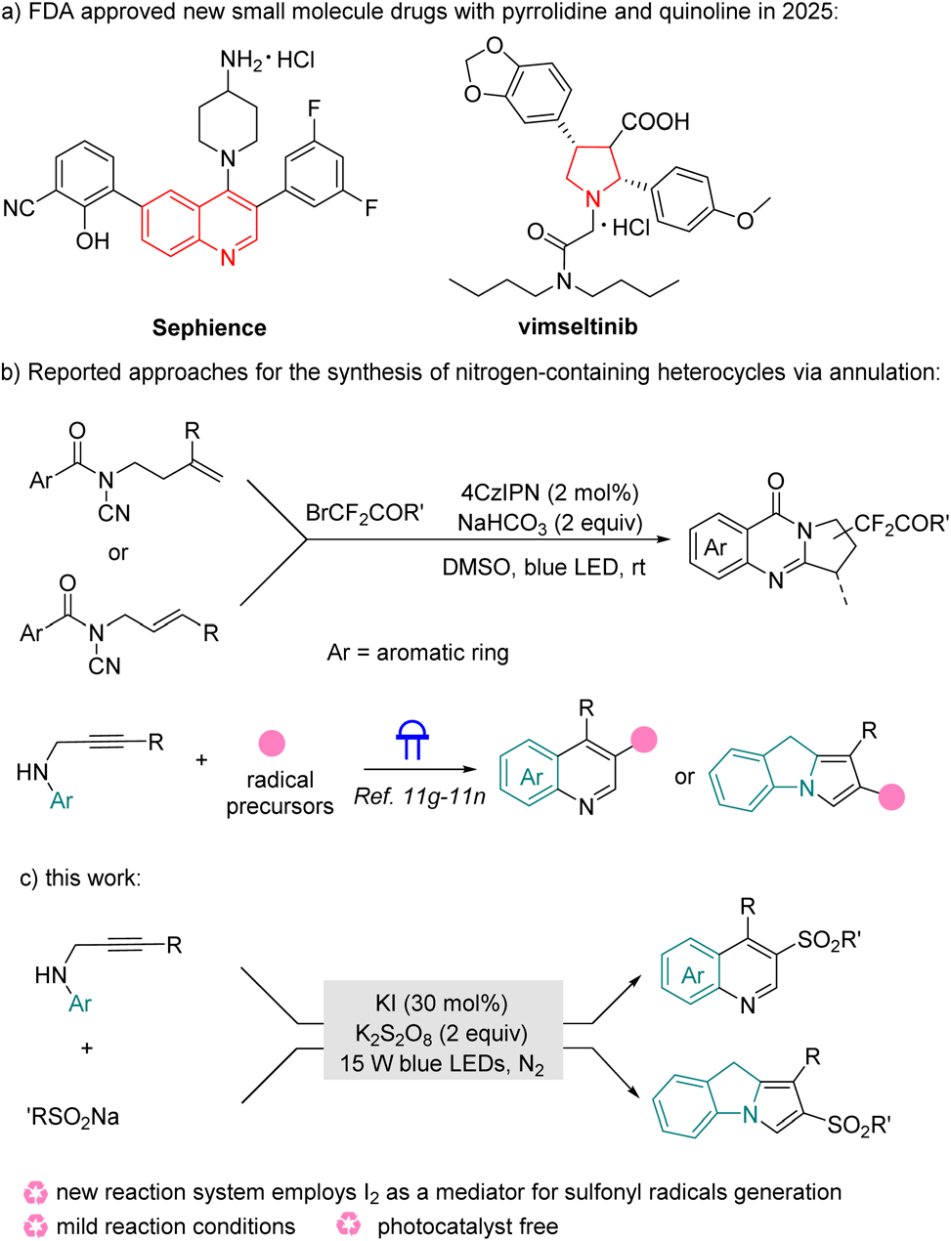
Background of this work.

To optimize the conditions for the proposed sulfonylation/cyclization cascade, we used the *N*-propargylamine 1a and 4-methylbenzenesulfinate derivative 2a as the template substrates (Table 1). The desired sulfonylated 6*H*-benzo[*c*]chromene 3a was isolated in 83% yield when the reaction was performed in the presence of KI (30 mol%) and K_2_S_2_O_8_ (2 equiv.) under 15 W blue LED irradiation in MeCN : H_2_O (2 : 1) (Table 1, entry 1). Subsequently, other oxidants were evaluated; however, all proved to be less effective than K_2_S_2_O_8_, yielding less of the desired product 3a (Table 1, entries 2–5). Additionally, attempts were made to replace 15 W blue LED with other light sources of different wavelengths, such as 425 nm, 390 nm, and 365 nm, but lower yields of product formation were observed (Table 1, entries 6–8). Control experiments showed that poor yields were observed in the absence of K_2_S_2_O_8_ and KI (Table 1, entries 9 and 10). Systematic evaluation of solvents revealed solvent-dependent reactivity: CH_3_CN (56%) DMF (37%), DMSO (25%), and THF (40%) showed inferior performance compared to mixed solvent (Table 1, entries 11–16). Therefore, we identified the optimal reaction conditions as the standard conditions: KI (30 mol%), K_2_S_2_O_8_ (2 equiv.), 15 W blue LED irradiation, MeCN : H_2_O (2 : 1), room temperature, 12 h.

**Table 1:** Optimization of the reaction conditions^a^

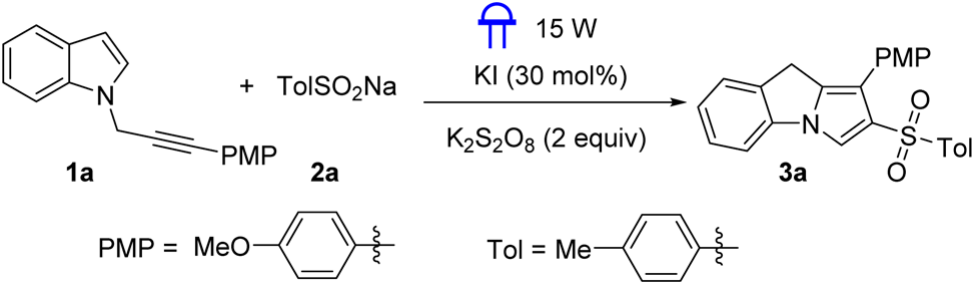		
Entry	Variation from standard conditions	Yield^b^ (%)
1	None	83
2	(NH_4_)_2_S_2_O_8_ instead of K_2_S_2_O_8_	48
3	Na_2_S_2_O_8_ instead of K_2_S_2_O_8_	75
4	Oxone instead of K_2_S_2_O_8_	Trace
5	TBHP instead of K_2_S_2_O_8_	Trace
6	425 nm	37
7	390 nm	30
8	365 nm	Trace
9	Without K_2_S_2_O_8_	Trace
10	Without KI	27
11	MeCN as solvent	56
12	DMSO as solvent	25
13	DMF as solvent	37
14	THF as solvent	40
15	1,4-Dioxane	35
16	Toluene	Trace

a Reaction conditions: 1a (0.2 mmol), 2a (0.4 mmol), K_2_S_2_O_8_ (2 equiv., 0.4 mmol), KI (30 mol%) in MeCN : H_2_O (2 : 1), at room temperature under nitrogen atmosphere, 12 h.

b Isolated yields.

After screening the optimized conditions, we proceeded to expand the reaction substrates. As depicted in Scheme 2, *N*-propargylamines with different substituents on the aromatic (Ar) ring were well tolerated, affording sulfonylated 9*H*-pyrrolo[1,2-*a*]indoles in moderate to excellent yields. For example, a *tert*-butyl substituted propargylamines was well compatible with this radical cascade reaction system, generating the desired product 3b in 82% yield. In addition, *N*-propargylamines with halogen on Ar ring were also well compatible with this sulfonylation/cyclization, affording the desired products 3e and 3f in 57 and 55% yields. Subsequently, we investigated the compatibility of the substituents on the aromatic ring in sodium sulfinates. Under standard conditions, sodium aryl sulfinates with alkyl, and halogen, substituents reacted well with *N*-propargylamine derivative 1h, providing products (3h–3l) in 69–85% yields. When the substrate contained a methyl group on the aromatic ring of indole, 3m was obtained in 61% yield.

**Scheme 2:**
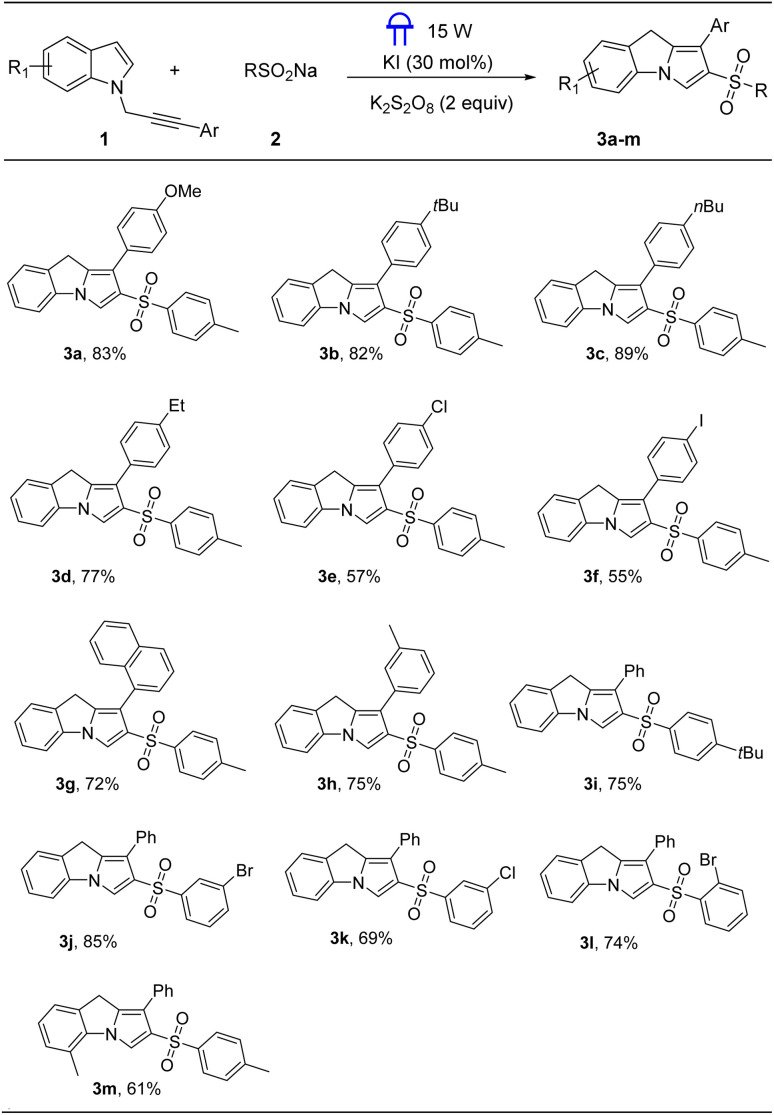
Substrates scope for the synthesis of 9*H*-pyrrolo[1,2-*a*]indoles^*a*^. ^*a*^ Reaction conditions: 1 (0.2 mmol), 2a (0.4 mmol), K_2_S_2_O_8_ (2 equiv., 0.4 mmol), KI (30 mol%) in MeCN : H_2_O (2 : 1), at room temperature under nitrogen atmosphere, 12 h; ^*b*^ isolated yields.

Subsequently, we evaluated the reaction scope of *N*-propargylamines with sodium sulfinates for the construction of sulfonylated quinolines under the optimized reaction conditions, and the results were shown in Scheme 3. To evaluate the functional group tolerance, a series of substituted sodium arylsulfinates were tested. Reaction of electron-donating groups on the para position of aromatic ring in aryl sulfinate derivatives proceeded smoothly to provide 5a and 5b in 78% and 56% yields, respectively. Aryl sulfinates bearing F, Cl, and Br functionalities were also compatible, delivering the desired sulfonylated quinolines (5e–5g) in 73–87% yields. Furthermore, the desired product 5h was obtained with 76% yield when sodium meta methylphenylsulfinate was served as sulfonylating precursor.

**Scheme 3:**
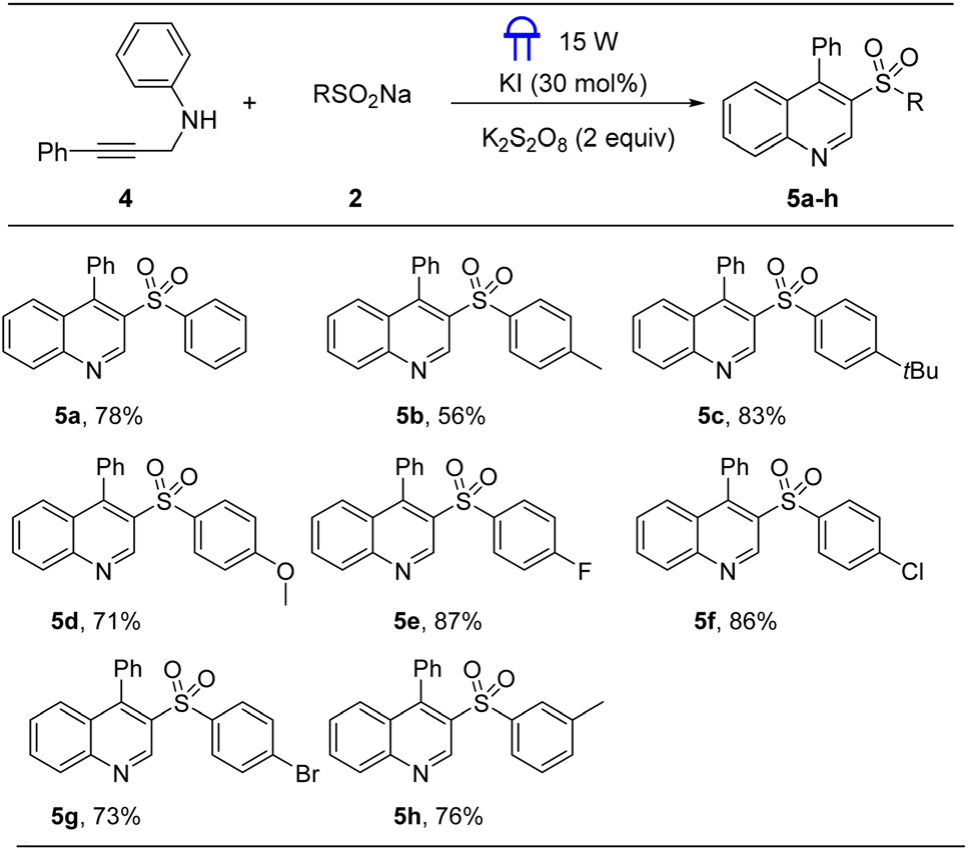
Substrate scope for the synthesis of quinolines^*a*^. ^*a*^ Reaction conditions: 1a (0.2 mmol), 2 (0.4 mmol), K_2_S_2_O_8_ (2 equiv., 0.4 mmol), KI (30 mol%) in MeCN : H_2_O (2 : 1), at room temperature under nitrogen atmosphere, 12 h; ^*b*^ isolated yields.

A control experiment (Scheme 4a) was conducted to elucidate the reaction mechanism. Under standard conditions, the presence of 2 equivalent of TEMPO (a radical quencher) completely inhibited the reaction, preventing the formation of the expected product. This result demonstrated that a radical pathway was involved in this approach. We propose a plausible reaction mechanism (Scheme 3b) that is consistent with our experimental data and previous reports.^11,12^ KI entered its catalytic cycle *via* oxidation by K_2_S_2_O_8_ to form I_2_. Under 15 W blue LED irradiation, this I_2_ was promoted to the excited state 
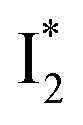
. This excited species, in conjunction with SO_4_^−^˙, facilitated the generation of sulfonyl radical A from sodium sulfinates. Subsequently, intermediate B was formed through the addition of radical intermediate A to the triple bond of the *N*-propargylamine 1a. Then, intermediate B added to indole C-2 position, affording intermediate C, which was oxidized by S_2_O_8_^2−^ to generate intermediate D through a SET (single electron transfer) process. Finally, intermediate D undergoes deprotonation and isomerization to obtain the final product 3a.

**Scheme 4:**
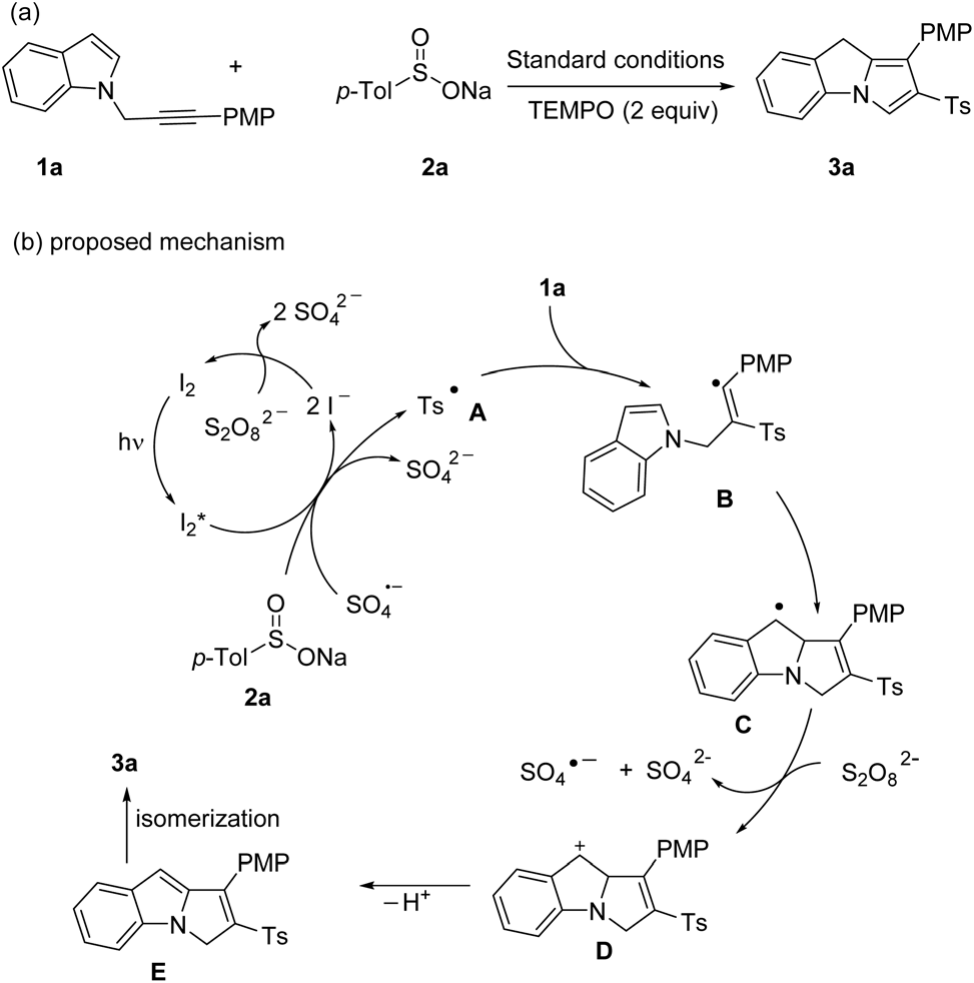
Control experiment and proposed mechanism.

## Conclusions

In summary, we have developed a photo-initiated efficient protocol for the synthesis of sulfonylated 9*H*-pyrrolo[1,2-*a*]indoles and quinolines quickly. This method enables the efficient radical cascade cyclization of *N*-propargylamines, offering significant advantages over conventional approaches, notably through sustainable visible light irradiation, operational simplicity, and photocatalyst-free conditions. Simultaneously, it also laid a certain foundation for exploring the green generation of sulfonyl radical from sodium sulfinates. The proposed mechanism covers a series of processes, including sulfonyl radical induced addition, annulation, and deprotonation. Building on our prior work, we are exploring further transformations of *N*-propargylamines.

## Conflicts of interest

There are no conflicts to declare.

## Supplementary Material

RA-016-D6RA01687A-s001

## Data Availability

The data underlying this study are available in the published article and its supplementary information (SI). Supplementary information is available. See DOI: https://doi.org/10.1039/d6ra01687a.
