# Supplementary material for: Visible-light promoted cascade annulation of N-propargylamines with sodium sulfinates to access sulfonylated 9H-pyrrolo[1,2-a]indoles and quinolines
Source: RSC Adv. 2026 Apr 29;16(24):22248–51. doi: 10.1039/d6ra01687a (PMC13127261; doi:10.1039/d6ra01687a)
Supplement: RA-016-D6RA01687A-s001 [file RA-016-D6RA01687A-s001.pdf]

Supplementary Information (SI) for RSC Advances.
This journal is © The Royal Society of Chemistry 2026

Supporting Information for

Visible-light promoted cascade annulation of *N*-propargylamines with sodium sulfonates to access sulfonylated 9*H*-pyrrolo[1,2-*a*]indoles and quinolines

Junke Wu,[a] Hongqiang Dong,*[a] and Zhuo Li,*[b]

[a] *College of Agriculture, Tarim University, Alaer, 843300, China; e-mail: dhqzky@163.com.*

[b.] *Shandong Key Laboratory for Green Prevention and Control of Agricultural Pests, Institute of Plant Protection, Shandong Academy of Agricultural Sciences, Jinan 250100, China. e-mail: lizhuo0613@163.com*

Table of Contents for Supporting Information

1. General information……………………………………………..……………..…….S2
2. Preparation of the starting materials……….. …………………………………….S3
3. General procedure for the synthesis of **3a** ..…………………………..…..........S3
4. Characterization data for the products………………………………………….…S4
5. References……………………………………………………….…....…….. …..…S15
6. Free radical-trapping experiment…………………………………………….…S16
7. NMR spectra of compounds..……………………………………………………S17

1. General considerations

All reactions were carried out under nitrogen atmosphere. ^{1}H NMR and ^{13}C NMR spectra were measured on a Bruker Avance NMR spectrometer (600 MHz/151 MHz/565 NMR or 400 MHz/101 MHz) in $CDCl_3$ as solvent and recorded in ppm relative to internal tetramethylsilane standard. Chemical shifts (δ) were reported in ppm, and coupling constants (J) were given in Hertz (Hz). Data were reported as s = singlet, d = doublet, t = triplet, q = quartet, dd = doublet of doublets, m = multiplet.

The Light Source and the Material of the Irradiation Vessel:

The photochemical reaction was carried out under visible light irradiation by a 15 W 380-390 nm purple LED at room temperature. This blue LED was purchased from taobao (link: https://baisilong.tmall.com/shop/view_shop.htm?spm=pc_detail.30350276.shop_block.dshopinfo.50b0238fQD32PG). The blue LED's energy peak wavelength is 460 nm. The reaction vessel is a borosilicate glass tube. The distance between the tube and lamp is about 3 cm, and no filter is applied.

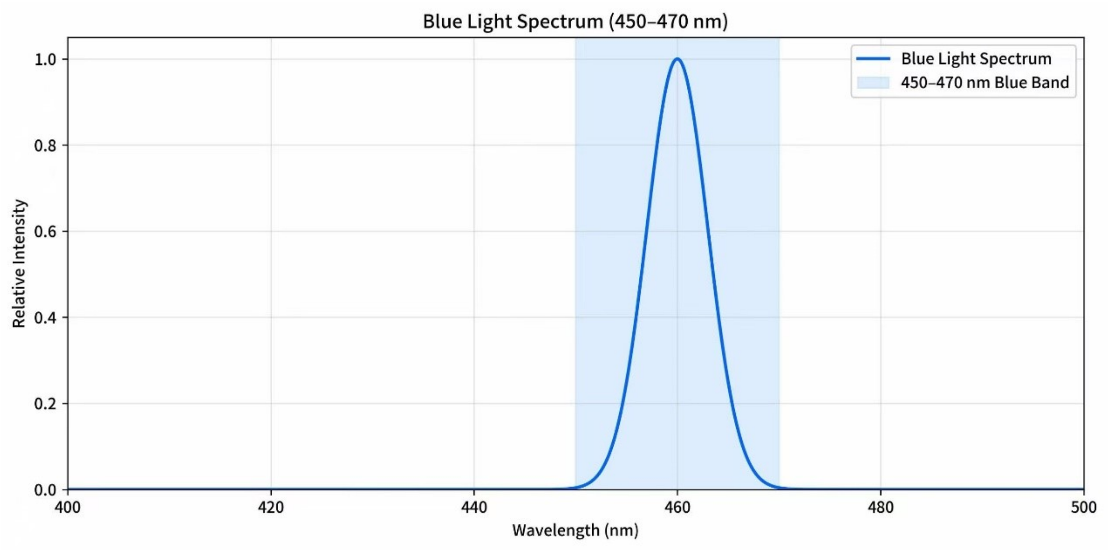

Figure S1. The spectral distribution of 15 W blue LED

2. Preparation of the starting materials

All N-propargylamines (**1a-1i** and **4**) and sodium sulfinates (**2**) were prepared according to the previous reported methods (S. Chen, P. Zhang, W. Shu, Y. Gao, G. Tang and Y. Zhao, *Org. Lett.*, 2016, **18**, 5712; M.-M. Zhang, Y. Sun, W. Wang, K. Chen, W.-C. Yang and L. Wang, *Org. Biomol. Chem.*, 2021, **19**, 3844).

3. General procedure for the synthesis of 3a

A dry 15 mL tube was charged with N-propargylamines (**1a**, 0.20 mmol), sodium sulfinates (**2a**, 0.40 mmol), CH_3CN/H_2O (2/1, 2 mL), KI (30 mol%), $K_2S_2O_8$ (2 equiv) and a magnetic stir bar. Then the mixture was stired under 15W blue led irradiation at room temperature in nitrogen atmosphere for 12 hours. After stirring for 12 h at room temperature, the mixture was extracted for 3 times with ethyl acetate (10 mL*3) and concentrated to obtain the crude product. Finally, the crude product was further purified by rapid chroma-tography (silica gel, petroleum ether (PE) / ethyl acetate (EA) = 10/1 – 5/1) to obtain the required product **3a**.

4. Characterization Data for Products

1-(4-methoxyphenyl)-2-tosyl-9*H*-pyrrolo[1,2-*a*]indole (3a) [1]

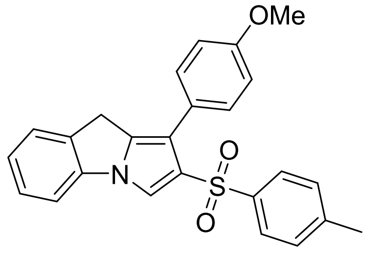

3a

(83% yield). ^{1}H NMR (600 MHz, $CDCl_3$) δ 7.90 (s, 1H), 7.47 (d, *J* = 8.0 Hz, 2H), 7.39 (dd, *J* = 13.4, 7.1 Hz, 3H), 7.35 (d, *J* = 8.4 Hz, 2H), 7.20 (t, *J* = 7.1 Hz, 1H), 7.08 (d, *J* = 8.0 Hz, 2H), 6.89 (d, *J* = 8.3 Hz, 2H), 3.86 (s, 3H), 3.83 (s, 2H), 2.32 (s, 3H). ^{13}C NMR (151 MHz, $CDCl_3$) δ 158.83 (s), 143.03 (s), 139.90 (s), 139.60 (s), 135.04 (s), 134.08 (s), 130.93 (s), 129.15 (s), 127.99 (s), 127.21 (s), 127.02 (s), 126.19 (s), 125.15 (s), 124.78 (s), 117.20 (s), 115.27 (s), 113.52 (s), 110.82 (s), 55.31 (s), 29.09 (s), 21.50 (s). The characterization data matched the literature.

1-(4-(*tert*-butyl)phenyl)-2-tosyl-9*H*-pyrrolo[1,2-*a*]indole (3b) [1]

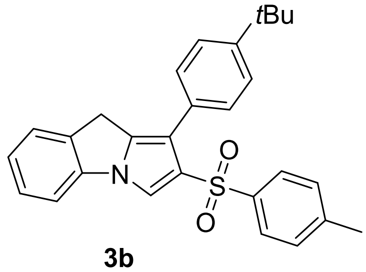

3b

(82% yield). ^{1}H NMR (600 MHz, $CDCl_3$) δ 7.91 (s, 1H), 7.43 (d, *J* = 8.1 Hz, 2H), 7.38 (dd, *J* = 13.8, 7.2 Hz, 3H), 7.33 (q, *J* = 8.3 Hz, 4H), 7.20 (t, *J* = 7.2 Hz, 1H), 7.02 (d, *J* = 8.1 Hz, 2H), 3.85 (s, 2H), 2.30 (s, 3H), 1.36

(s, 9H). ^{13}C NMR (151 MHz, $CDCl_3$) δ 150.14 (s), 142.95 (s), 139.65 (d, *J* = 16.3 Hz), 135.04 (s), 134.13 (s), 129.40 (d, *J* = 5.2 Hz), 129.01 (s), 127.98 (s), 127.32 (s), 127.18 (s), 126.19 (s), 125.14 (s), 124.95 (s), 117.44 (s), 115.28 (s), 110.82 (s), 34.60 (s), 31.43 (s), 29.19 (s), 21.49 (s).

1-(4-butylphenyl)-2-tosyl-9*H*-pyrrolo[1,2-*a*]indole (3c) [1]

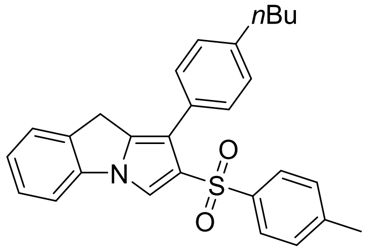

(89% yield). ^{1}H NMR (600 MHz, $CDCl_3$) δ 7.90 (s, 1H), 7.45 (d, *J* = 8.2 Hz, 2H), 7.37 (dd, *J* = 13.3, 7.4 Hz, 3H), 7.31 (d, *J* = 7.9 Hz, 2H), 7.19 (t, *J* = 7.3 Hz, 1H), 7.14 (d, *J* = 7.9 Hz, 2H), 7.04 (d, *J* = 8.1 Hz, 2H), 3.82 (s, 2H), 2.64 (t, *J* = 7.7 Hz, 2H), 2.30 (s, 3H), 1.70 – 1.60 (m, 2H), 1.39 (dd, *J* = 14.9, 7.4 Hz, 2H), 0.97 (t, *J* = 7.4 Hz, 3H). ^{13}C NMR (151 MHz, $CDCl_3$) δ 142.99 (s), 141.90 (s), 139.79 (s), 139.58 (s), 135.08 (s), 134.10 (s), 129.61 (d, *J* = 11.9 Hz), 129.06 (s), 128.16 (s), 127.98 (s), 127.30 (s), 127.09 (s), 126.18 (s), 125.15 (s), 117.52 (s), 115.34 (s), 110.82 (s), 35.41 (s), 33.68 (s), 29.16 (s), 22.33 (s), 21.49 (s), 14.07 (s).

1-(4-ethylphenyl)-2-tosyl-9*H*-pyrrolo[1,2-*a*]indole (3d) [1]

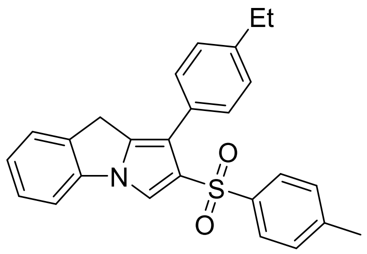

(77% yield). ^{1}H NMR (600 MHz, $CDCl_3$) δ 7.82 (s, 1H), 7.38 (d, *J* = 8.1 Hz, 2H), 7.30 (dd, *J* = 13.7, 7.2 Hz, 3H), 7.24 (d, *J* = 7.8 Hz, 2H), 7.10 (dd, *J* = 17.1, 7.6 Hz, 3H), 6.97 (d, *J* = 8.1 Hz, 2H), 3.75 (s, 2H), 2.60 (q, *J* = 7.6 Hz, 2H), 2.22 (s, 3H), 1.20 (t, *J* = 7.6 Hz, 3H). ^{13}C NMR (151 MHz, $CDCl_3$) δ 143.30 (s), 143.00 (s), 139.88 (s), 139.58 (s), 135.11 (s), 134.10 (s), 129.68 (d, *J* = 6.8 Hz), 129.10 (s), 127.98 (s), 127.59 (s), 127.27 (s), 127.08 (s), 126.19 (s), 125.16 (s), 117.53 (s), 115.37 (s), 110.82 (s), 29.16 (s), 28.68 (s), 21.50 (s), 15.71 (s).

1-(4-chlorophenyl)-2-tosyl-9*H*-pyrrolo[1,2-*a*]indole (3e) [1]

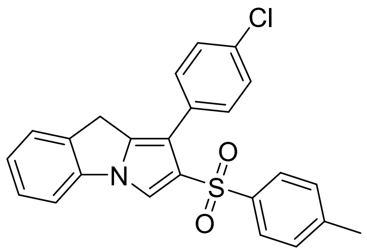

(57% yield). ^{1}H NMR (600 MHz, $CDCl_3$) δ 7.85 (s, 1H), 7.41 (d, *J* = 8.1 Hz, 2H), 7.35 – 7.29 (m, 5H), 7.24 (d, *J* = 8.3 Hz, 2H), 7.15 (t, *J* = 7.0 Hz, 1H), 7.03 (d, *J* = 8.0 Hz, 2H), 3.77 (s, 2H), 2.26 (s, 3H). ^{13}C NMR (151 MHz, $CDCl_3$) δ 143.30 (s), 139.69 (s), 139.46 (s), 135.48 (s), 133.85 (s), 133.17 (s), 131.01 (s), 129.26 (s), 128.31 (s), 128.12 (s), 127.19 (s), 127.03

(s), 126.22 (s), 125.37 (s), 116.27 (s), 115.70 (s), 110.92 (s), 29.16 (s), 21.51 (s). The characterization data matched the literature.

1-(4-iodophenyl)-2-tosyl-9*H*-pyrrolo[1,2-*a*]indole (3f) [1]

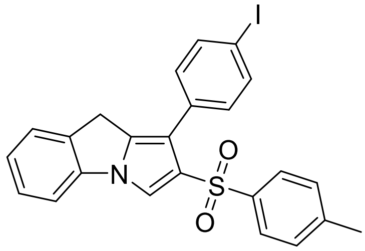

(55% yield). ^{1}H NMR (600 MHz, $CDCl_3$) δ 7.92 (s, 1H), 7.67 (d, J = 8.0 Hz, 2H), 7.49 (d, J = 8.0 Hz, 2H), 7.41 (dd, J = 13.3, 6.8 Hz, 3H), 7.23 (d, J = 7.1 Hz, 1H), 7.18 (d, J = 7.9 Hz, 2H), 7.11 (d, J = 8.0 Hz, 2H), 3.84 (s, 2H), 2.34 (s, 3H). ^{13}C NMR (151 MHz, $CDCl_3$) δ 143.32 (s), 139.69 (s), 139.44 (s), 137.25 (s), 135.43 (s), 133.84 (s), 132.11 (s), 131.54 (s), 129.28 (s), 128.12 (s), 127.19 (s), 126.95 (s), 126.23 (s), 125.39 (s), 116.35 (s), 115.79 (s), 110.92 (s), 92.92 (s), 29.20 (s), 21.52 (s). The characterization data matched the literature.

1-(naphthalen-1-yl)-2-tosyl-9*H*-pyrrolo[1,2-*a*]indole (3g) [1]

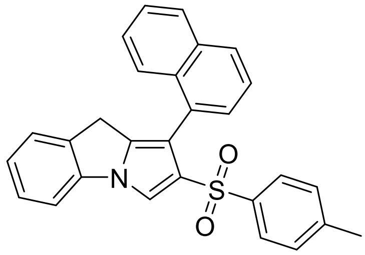

(72% yield). ^{1}H NMR (600 MHz, $CDCl_3$) δ 7.97 (s, 1H), 7.78 (d, J = 7.9

Hz, 1H), 7.74 (d, J = 8.2 Hz, 1H), 7.44 – 7.38 (m, 3H), 7.33 (t, J = 7.7 Hz, 1H), 7.28 (t, J = 8.0 Hz, 2H), 7.13 (t, J = 7.5 Hz, 1H), 7.02 (dd, J = 13.6, 8.2 Hz, 4H), 6.59 (d, J = 8.0 Hz, 2H), 3.50 (d, J = 6.0 Hz, 2H), 2.02 (s, 3H). ^{13}C NMR (151 MHz, $CDCl_3$) δ 141.62 (s), 138.71 (s), 137.67 (s), 135.12 (s), 133.25 (s), 132.24 (s), 131.21 (s), 128.69 (s), 128.13 (s), 127.81 (s), 127.56 (s), 127.16 (s), 127.00 (s), 126.23 (s), 125.22 (s), 124.62 (s), 124.36 – 124.01 (m), 113.69 (s), 113.17 (s), 109.88 (s), 27.90 (s), 20.18 (s). The characterization data matched the literature.

1-(*m*-tolyl)-2-tosyl-9*H*-pyrrolo[1,2-*a*]indole (3h) [1]

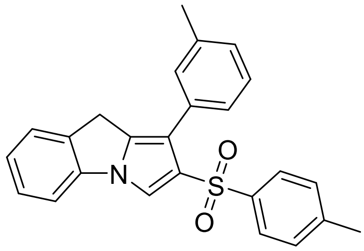

(75% yield). ^{1}H NMR (600 MHz, $CDCl_3$) δ 7.84 (s, 1H), 7.41 (d, J = 8.2 Hz, 2H), 7.29 (t, J = 10.0 Hz, 3H), 7.16 – 7.09 (m, 3H), 7.05 (s, 1H), 7.01 (dd, J = 13.5, 7.8 Hz, 3H), 3.74 (s, 2H), 2.24 (s, 3H), 2.23 (s, 3H). ^{13}C NMR (151 MHz, $CDCl_3$) δ 141.98 (s), 138.84 (s), 138.48 (s), 136.43 (s), 134.14 (s), 133.01 (s), 131.28 (s), 129.31 (s), 128.03 (s), 126.92 (d, J = 3.8 Hz), 126.28 (s), 126.00 (s), 125.65 (s), 125.12 (s), 124.12 (s), 116.51 (s), 114.38 (s), 109.78 (s), 28.09 (s), 20.39 (d, J = 11.2 Hz). The characterization data matched the literature.

2-((4-(*tert*-butyl)phenyl)sulfonyl)-1-phenyl-9*H*-pyrrolo[1,2-*a*]indole (3i) [1]

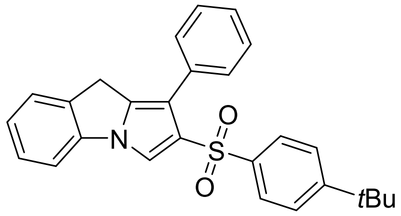

(75% yield). ^{1}H NMR (600 MHz, $CDCl_3$) δ 7.93 (s, 1H), 7.49 (d, *J* = 8.2 Hz, 2H), 7.40 (t, *J* = 8.2 Hz, 5H), 7.32 (dd, *J* = 15.4, 7.8 Hz, 3H), 7.26 (d, *J* = 7.7 Hz, 2H), 7.21 (t, *J* = 7.3 Hz, 1H), 3.84 (s, 2H), 1.25 (s, 9H). ^{13}C NMR (151 MHz, $CDCl_3$) δ 156.03 (s), 139.57 (s), 135.24 (s), 134.04 (s), 132.49 (s), 129.80 (s), 128.04 (d, *J* = 4.4 Hz), 127.22 (s), 127.08 (s), 126.19 (s), 125.46 (s), 125.21 (s), 117.55 (s), 115.38 (s), 110.86 (s), 35.00 (s), 31.04 (s), 29.12 (s). The characterization data matched the literature.

2-((3-bromophenyl)sulfonyl)-1-phenyl-9*H*-pyrrolo[1,2-*a*]indole (3j) [1]

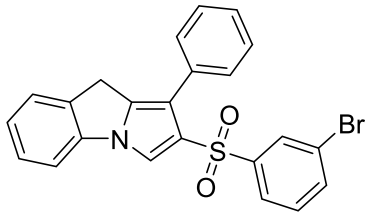

(85% yield). ^{1}H NMR (600 MHz, $CDCl_3$) δ 7.94 (s, 1H), 7.63 (s, 1H), 7.50 (d, *J* = 7.9 Hz, 1H), 7.47 (d, *J* = 7.9 Hz, 1H), 7.44 – 7.33 (m, 9H), 7.22 (t, *J* = 7.4 Hz, 1H), 7.11 (t, *J* = 7.9 Hz, 1H), 3.83 (s, 2H). ^{13}C NMR (151 MHz, $CDCl_3$) δ 144.38 (s), 139.41 (s), 135.53 (s), 135.34 (s), 134.06 (s), 132.07 (s), 130.34 (s), 130.00 (s), 129.81 (s), 128.25 (s), 128.08 (s), 127.66 (s), 126.27 (s), 126.08 (s), 125.75 (s), 125.46 (s), 122.43 (s), 117.62 (s), 115.70

(s), 111.00 (s), 29.04 (s). The characterization data matched the literature.

2-((3-chlorophenyl)sulfonyl)-1-phenyl-9*H*-pyrrolo[1,2-*a*]indole (3k)[1]

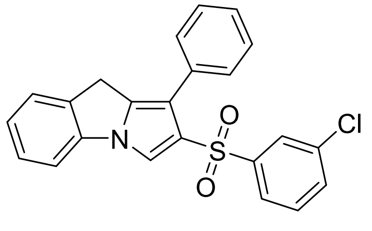

(69% yield). ^{1}H NMR (600 MHz, $CDCl_3$) δ 7.95 (s, 1H), 7.48 (s, 1H), 7.41 (d, J = 5.9 Hz, 3H), 7.36 (dd, J = 12.6, 5.6 Hz, 7H), 7.23 (t, J = 7.4 Hz, 1H), 7.18 (t, J = 7.9 Hz, 1H), 3.85 (s, 2H). ^{13}C NMR (151 MHz, $CDCl_3$) δ 144.23 (s), 139.43 (s), 135.51 (s), 134.61 (s), 134.06 (s), 132.42 (s), 132.08 (s), 129.78 (d, J = 9.5 Hz), 128.23 (s), 128.09 (s), 127.57 (d, J = 15.3 Hz), 126.27 (s), 126.10 (s), 125.46 (s), 125.30 (s), 117.64 (s), 115.70 (s), 110.99 (s), 29.05 (s). The characterization data matched the literature.

2-((2-bromophenyl)sulfonyl)-1-phenyl-9*H*-pyrrolo[1,2-*a*]indole (3l) [1]

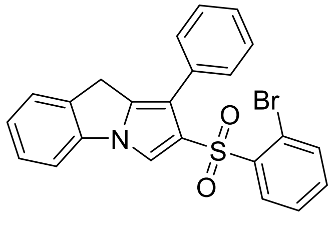

(74% yield). ^{1}H NMR (600 MHz, $CDCl_3$) δ 8.10 (s, 1H), 7.74 (d, J = 7.9 Hz, 1H), 7.52 (d, J = 7.8 Hz, 1H), 7.45 (d, J = 7.8 Hz, 1H), 7.42 (t, J = 7.1 Hz, 2H), 7.22 (dd, J = 12.7, 7.3 Hz, 7H), 7.09 (t, J = 7.7 Hz, 1H), 3.85 (s, 2H). ^{13}C NMR (151 MHz, $CDCl_3$) δ 140.22 (s), 139.60 (s), 134.87 (s),

134.70 (s), 134.18 (s), 133.60 (s), 132.02 (s), 131.69 (s), 129.64 (s), 128.06 (s), 127.92 (s), 127.21 (s), 126.93 (s), 126.28 (s), 125.34 (s), 124.45 (s), 120.71 (s), 117.89 (s), 117.51 (s), 111.05 (s), 28.97 (s).

5-methyl-1-phenyl-2-tosyl-9*H*-pyrrolo[1,2-*a*]indole (3m) [1]

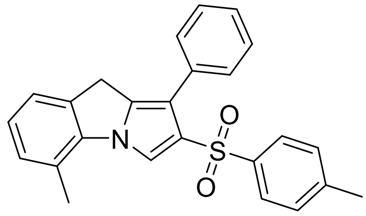

(61% yield). ^{1}H NMR (600 MHz, $CDCl_3$) δ 7.98 (s, 1H), 7.19 – 7.15 (m, 2H), 7.08 (d, J = 7.6 Hz, 1H), 7.04 (t, J = 7.4 Hz, 1H), 6.98 (d, J = 8.0 Hz, 2H), 3.77 (s, 2H), 2.59 (s, 3H), 2.24 (s, 3H). ^{13}C NMR (151 MHz, $CDCl_3$) δ 143.00 (s), 139.85 (s), 138.53 (s), 135.80 (s), 134.21 (s), 132.50 (s), 131.53 (s), 130.22 (s), 129.82 (s), 129.08 (s), 128.07 (s), 127.21 (s), 126.83 (s), 125.10 (s), 123.70 (s), 122.86 (s), 118.17 (s), 117.04 (s), 29.01 (s), 21.48 (s), 18.47 (s). The characterization data matched the literature.

4-phenyl-3-(phenylsulfonyl)quinoline (5a) [2]

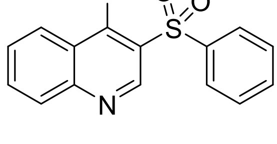

(78% yield). ^{1}H NMR (600 MHz, $CDCl_3$) δ 9.74 (s, 1H), 8.15 (d, J = 8.5 Hz, 1H), 7.75 (t, J = 7.6 Hz, 1H), 7.38 (t, J = 8.9 Hz, 3H), 7.28 – 7.24 (m, 5H), 7.20 – 7.17 (m, 2H), 6.86 (d, J = 7.7 Hz, 2H). ^{13}C NMR (151 MHz,

$CDCl_3$) δ 150.06 (s), 149.83 (s), 147.72 (s), 140.87 (s), 133.02 (s), 132.52 (s), 132.29 (s), 130.05 (s), 129.71 (s), 129.71, 129.17, 128.73, 128.70, 127.90, 127.88, 127.74 (s), 127.45 (s). The characterization data matched the literature.

4-phenyl-3-tosylquinoline (5b) [2]

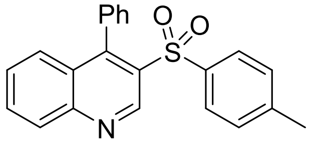

(56% yield). ^{1}H NMR (600 MHz, $CDCl_3$) δ 9.72 (s, 1H), 8.14 (d, *J* = 8.5 Hz, 1H), 7.80 – 7.69 (m, 1H), 7.39 (q, *J* = 7.2 Hz, 2H), 7.27 (t, *J* = 7.7 Hz, 3H), 7.14 (d, *J* = 8.3 Hz, 2H), 6.98 (d, *J* = 8.2 Hz, 2H), 6.89 (d, *J* = 7.1 Hz, 2H), 2.28 (s, 3H). ^{13}C NMR (151 MHz, $CDCl_3$) δ 149.79 (d, *J* = 16.8 Hz), 147.82 (s), 144.04 (s), 138.01 (s), 132.67 (s), 132.16 (s), 130.05 (s), 129.69 (s), 129.26 (s), 128.67 (s), 127.97 (s), 127.83 (s), 127.66, 127.54, 127.45, 21.58 (s).

3-((4-(*tert*-butyl)phenyl)sulfonyl)-4-phenylquinoline (5c) [2]

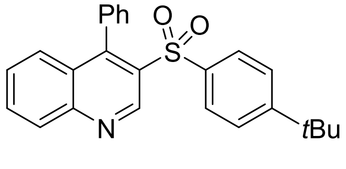

(83% yield). ^{1}H NMR (400 MHz, $CDCl_3$) δ 9.71 (s, 1H), 8.12 (d, *J* = 8.4 Hz, 1H), 7.72 (ddd, *J* = 8.3, 6.8, 1.3 Hz, 1H), 7.35 (dd, *J* = 11.8, 4.4 Hz, 2H), 7.23 (dd, *J* = 12.4, 4.3 Hz, 3H), 7.16 (s, 4H), 6.88 – 6.82 (m, 2H), 1.19

(s, 9H). ^{13}C NMR (101 MHz, $CDCl_3$) δ 156.98 (s), 149.74 (s), 147.68 (s), 137.74 (s), 132.73 (s), 132.15 (s), 130.04, 129.68, 128.59, 127.71, 127.44, 125.67 (s), 35.14 (s), 31.04 (s).

3-((4-methoxyphenyl)sulfonyl)-4-phenylquinoline (5d) [2]

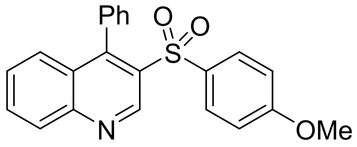

(71% yield). ^{1}H NMR (400 MHz, $CDCl_3$) δ 9.71 (s, 1H), 8.13 (d, *J* = 8.4 Hz, 1H), 7.74 (ddd, *J* = 8.4, 6.8, 1.4 Hz, 1H), 7.43 – 7.35 (m, 2H), 7.32 – 7.23 (m, 3H), 7.16 (d, *J* = 9.0 Hz, 2H), 6.94 – 6.89 (m, 2H), 6.66 – 6.61 (m, 2H), 3.73 (s, 3H). ^{13}C NMR (101 MHz, $CDCl_3$) δ 163.27 (s), 149.66 (d, *J* = 4.9 Hz), 147.78 (s), 132.92, 132.76, 132.46, 132.10 (s), 130.17, 130.09, 129.67 (s), 128.69 (s), 127.81, 127.72, 127.54, 127.42, 113.89 (s), 55.67 (s).

3-((4-fluorophenyl)sulfonyl)-4-phenylquinoline (5e) [2]

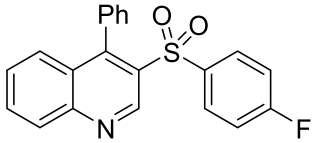

(87% yield). ^{1}H NMR (400 MHz, $CDCl_3$) δ 9.70 (s, 1H), 8.12 (d, *J* = 8.4 Hz, 1H), 7.74 (ddd, *J* = 8.3, 6.9, 1.3 Hz, 1H), 7.43 – 7.35 (m, 2H), 7.29 – 7.18 (m, 5H), 6.90 – 6.78 (m, 4H). ^{13}C NMR (101 MHz, $CDCl_3$) δ δ 165.27 (d, *J* = 254.6 Hz), 149.90 (s), 147.46 (s), 136.84 (s), 132.41, 132.22,

130.78 (s), 130.10 (s), 129.30 (d, J = 85.2 Hz), 127.92 (d, J = 19.3 Hz), 127.41 (s), 115.92 (d, J = 22.4 Hz). The characterization data matched the literature.

3-((4-chlorophenyl)sulfonyl)-4-phenylquinoline (5f) [2]

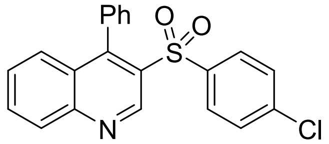

(86% yield). ^{1}H NMR (400 MHz, $CDCl_3$) δ 9.71 (s, 1H), 8.14 (d, J = 8.5 Hz, 1H), 7.76 (t, J = 7.6 Hz, 1H), 7.40 (dd, J = 13.3, 6.7 Hz, 2H), 7.27 (dd, J = 12.8, 5.4 Hz, 3H), 7.17 – 7.10 (m, 4H), 6.88 (d, J = 7.5 Hz, 2H). ^{13}C NMR (101 MHz, $CDCl_3$) δ 150.00, 149.92, 147.47 (s), 139.76 (s), 139.30 (s), 132.46 (s), 132.07 (s), 130.11 (s), 129.75 (s), 129.33 (s), 128.91 (s), 128.05 (s), 127.83 (s), 127.42, 127.39. The characterization data matched the literature.

3-((4-bromophenyl)sulfonyl)-4-phenylquinoline (5g) [2]

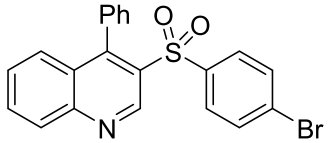

(73% yield). ^{1}H NMR (400 MHz, $CDCl_3$) δ 9.70 (s, 1H), 8.13 (d, J = 8.5 Hz, 1H), 7.75 (dd, J = 11.2, 4.0 Hz, 1H), 7.39 (dd, J = 14.2, 6.9 Hz, 2H), 7.32 – 7.24 (m, 5H), 7.07 (d, J = 8.5 Hz, 2H), 6.88 (d, J = 7.2 Hz, 2H). ^{13}C NMR (101 MHz, $CDCl_3$) δ 149.99, 149.90, 147.43 (s), 139.80 (s), 132.48,

132.42, 132.00, 131.88, 130.09, 129.74, 129.37, 128.91, 128.33, 128.05, 127.83 (s), 127.42, 127.37.

4-phenyl-3-(*m*-tolylsulfonyl)quinoline (5h) [2]

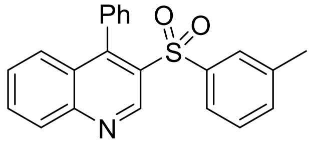

(76% yield). ^{1}H NMR (400 MHz, $CDCl_3$) δ 9.74 (s, 1H), 8.15 (d, *J* = 8.4 Hz, 1H), 7.75 (ddd, *J* = 8.4, 6.8, 1.4 Hz, 1H), 7.38 (tdd, *J* = 3.9, 3.2, 1.8 Hz, 2H), 7.29 – 7.23 (m, 3H), 7.20 – 7.16 (m, 1H), 7.09 (dd, *J* = 4.8, 1.6 Hz, 2H), 6.98 (s, 1H), 6.87 (dd, *J* = 8.0, 1.0 Hz, 2H), 2.16 (s, 3H). ^{13}C NMR (101 MHz, $CDCl_3$) δ 149.92, 149.78, 147.68 (s), 140.56 (s), 138.73 (s), 133.87 (s), 132.58, 132.49, 132.21 (s), 130.11 (s), 129.69 (s), 128.57, 128.43, 127.84 (s), 127.60, 127.43, 125.03 (s), 21.11 (s).

5. References

[1] X. Xie, P. Li and L. Wang, *Eur. J. Org. Chem.*, 2019, 221.

[2] Y. Zhang, W. Chen, X. Jia, L. Wang and P. Li, *Chem. Commun.*, 2019, **55**, 2785-2788.

6. Free radical-trapping experiment

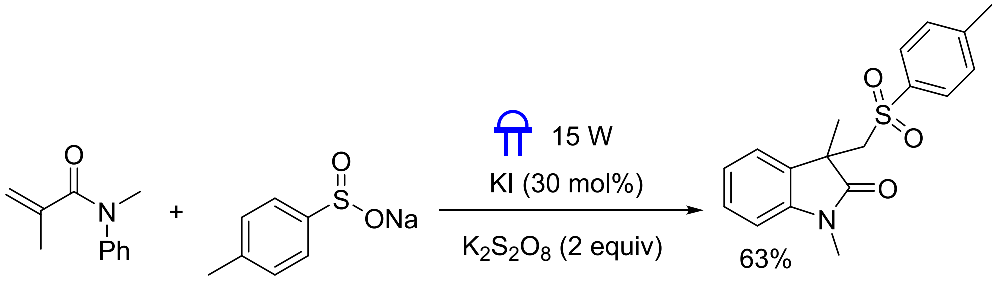

Replacing N-propargylamines with N-arylacrylamide, a classic radical acceptor frequently employed in radical tandem reactions, delivered the sulfonylated oxindole in 63% yield. This provides strong evidence for a free radical mechanism.

^{1}H NMR (600 MHz, $CDCl_3$) δ 7.37 (d, J = 8.2 Hz, 2H), 7.31 – 7.26 (m, 1H), 7.16 (d, J = 8.0 Hz, 2H), 7.08 (d, J = 7.2 Hz, 1H), 6.92 (t, J = 7.5 Hz, 1H), 6.84 (d, J = 7.8 Hz, 1H), 3.85 (d, J = 14.6 Hz, 1H), 3.67 (d, J = 14.6 Hz, 1H), 3.16 (s, 3H), 2.39 (s, 3H), 1.38 (s, 3H).

7. NMR spectra of compounds

^{1}H NMR of **3a** in $CDCl_3$ (600 MHz, $CDCl_3$)

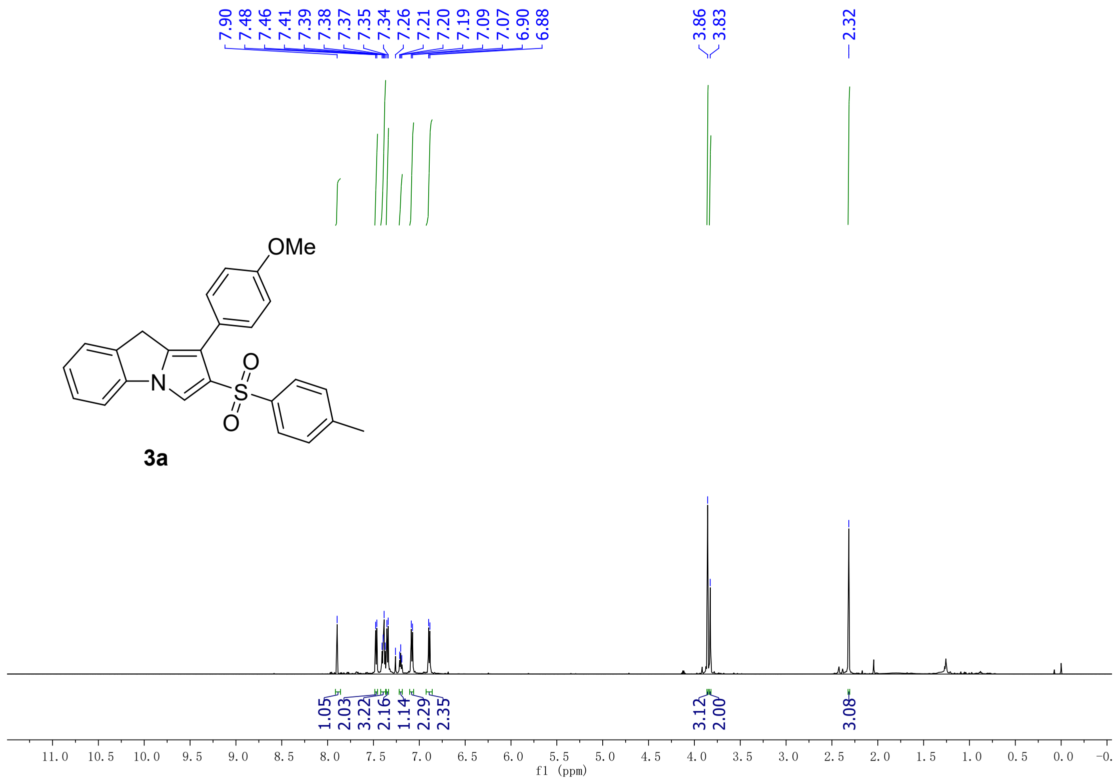

^{13}C NMR of **3a** in $CDCl_3$ (151 MHz, $CDCl_3$)

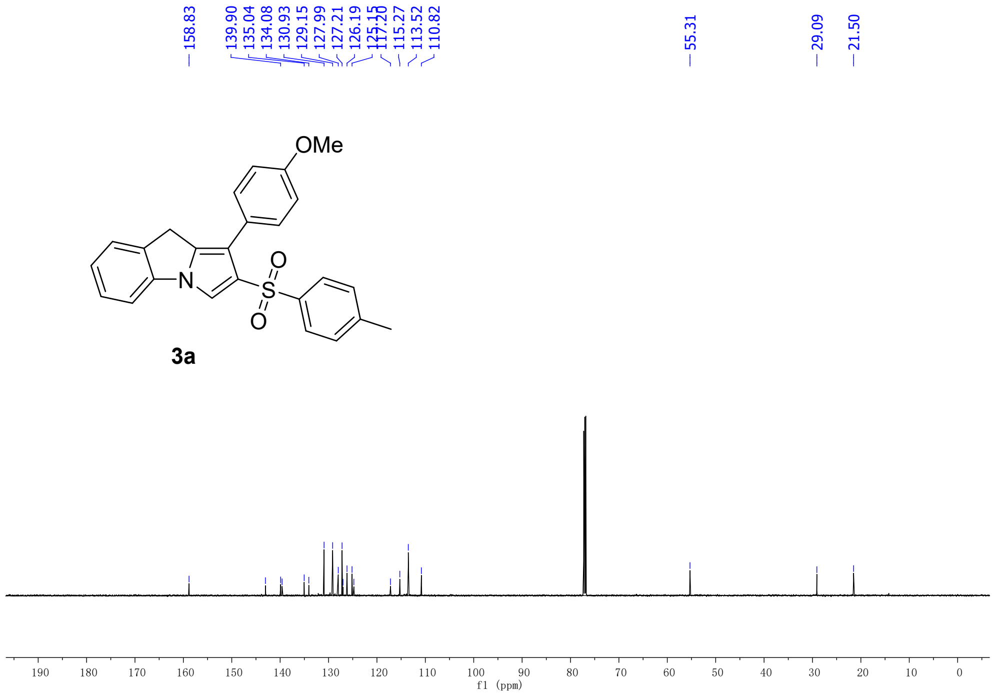

^{1}H NMR of **3b** in $CDCl_3$ (600 MHz, $CDCl_3$)

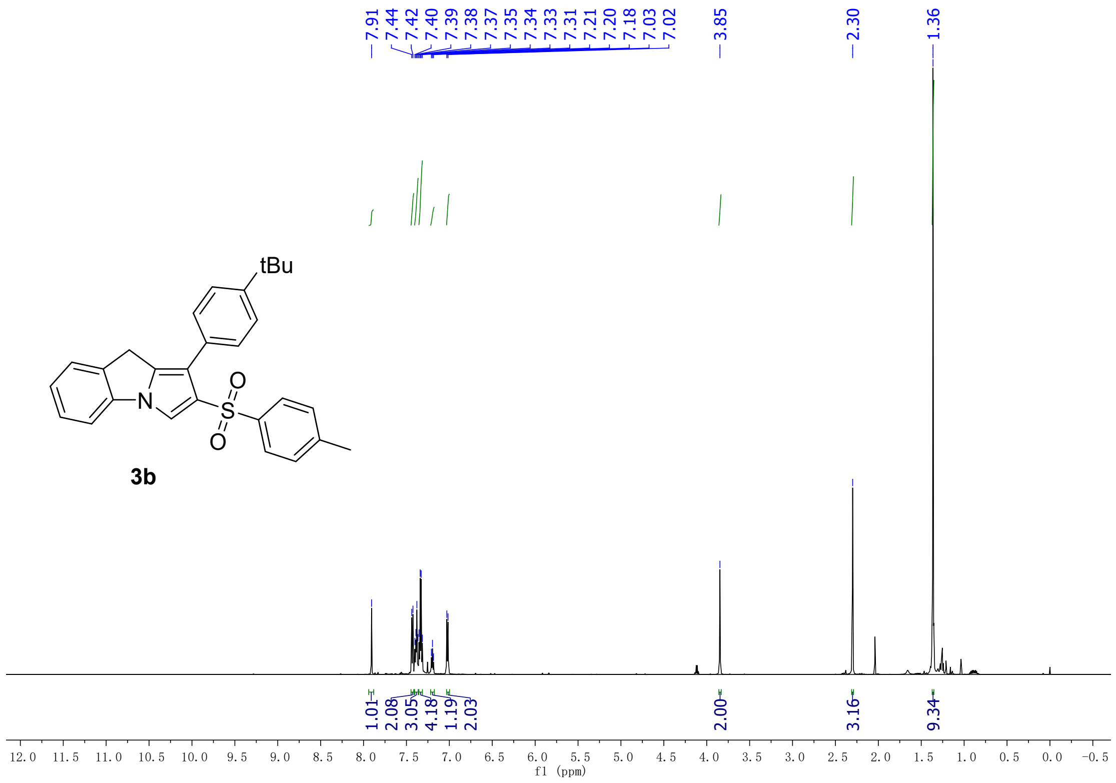

^{13}C NMR of **3b** in $CDCl_3$ (151 MHz, $CDCl_3$)

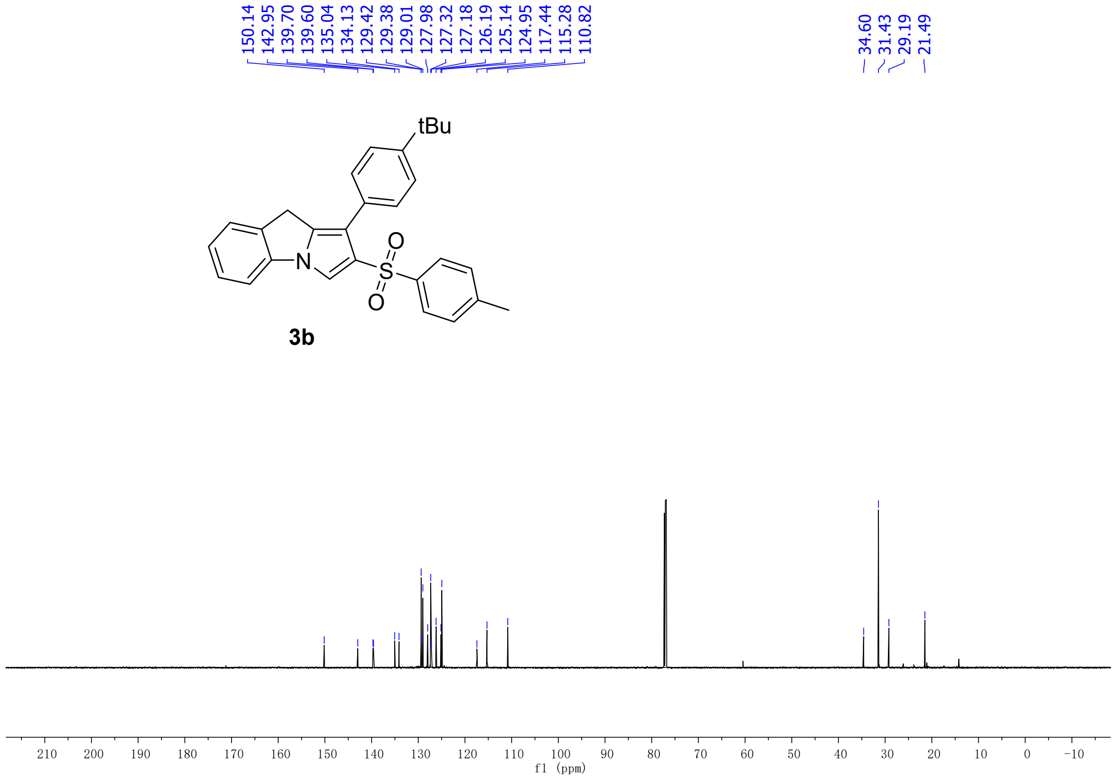

^{1}H NMR of **3c** in $CDCl_3$ (600 MHz, $CDCl_3$)

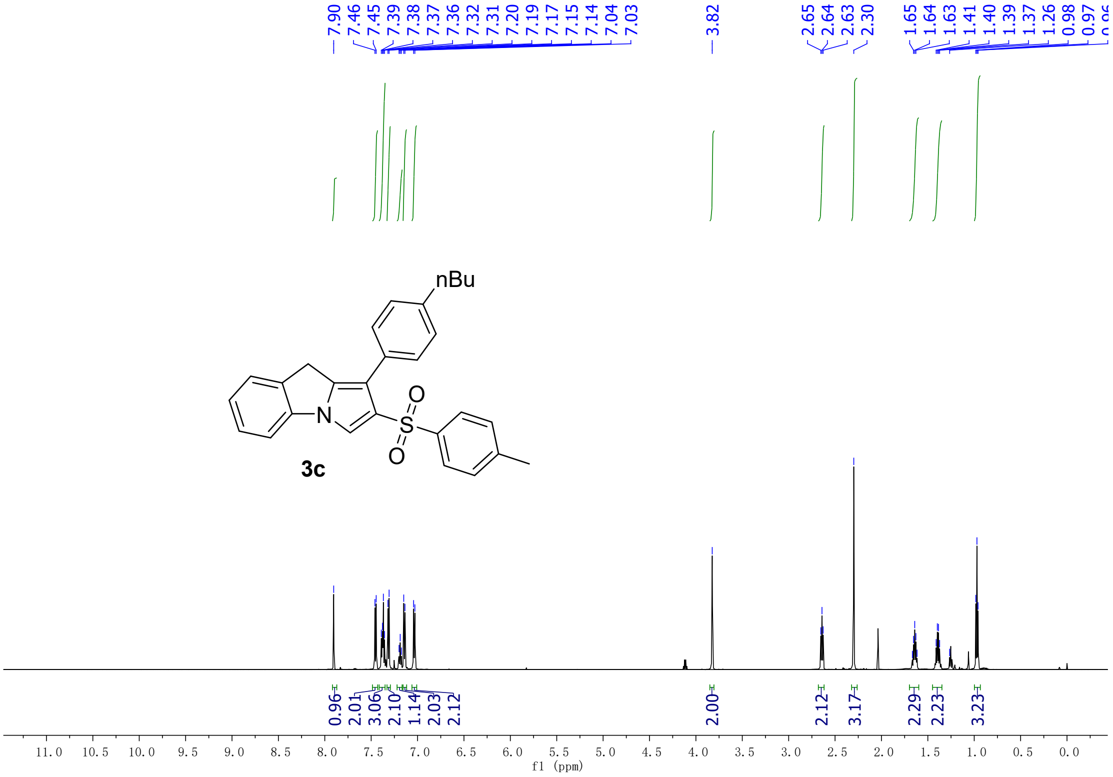

^{13}C NMR of **3c** in $CDCl_3$ (151 MHz, $CDCl_3$)

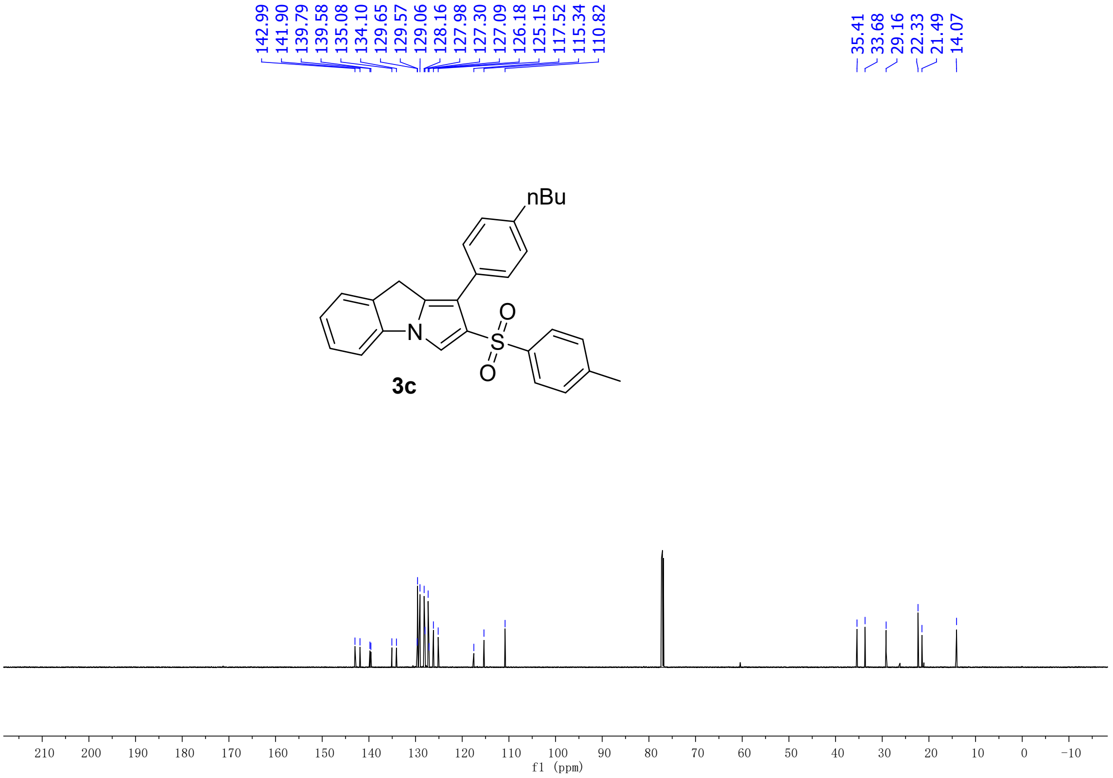

^{1}H NMR of **3d** in $CDCl_3$ (600 MHz, $CDCl_3$)

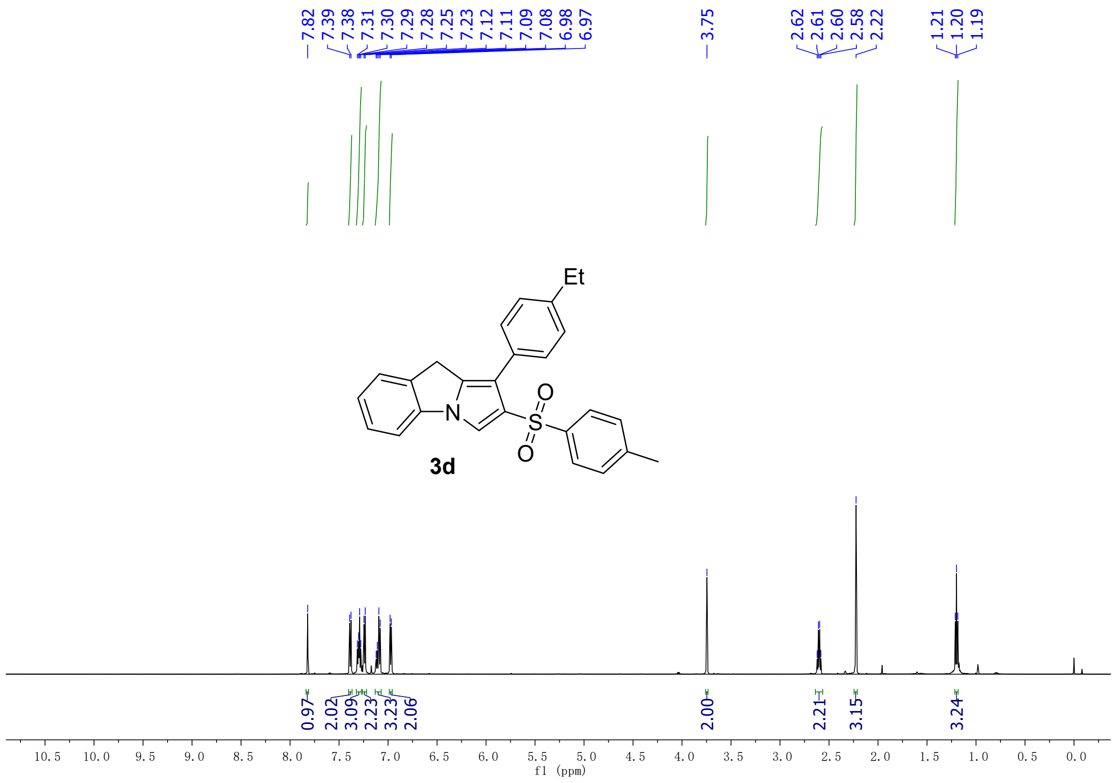

^{13}C NMR of **3d** in $CDCl_3$ (151 MHz, $CDCl_3$)

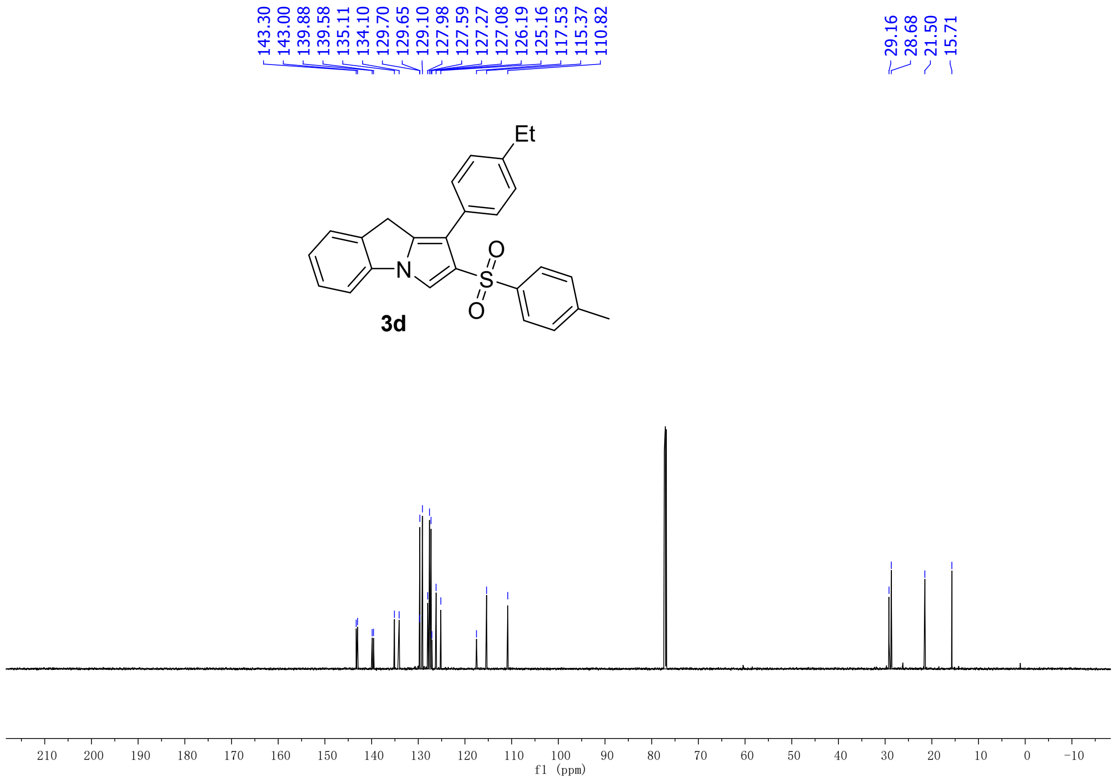

^{1}H NMR of **3e** in $CDCl_3$ (600 MHz, $CDCl_3$)

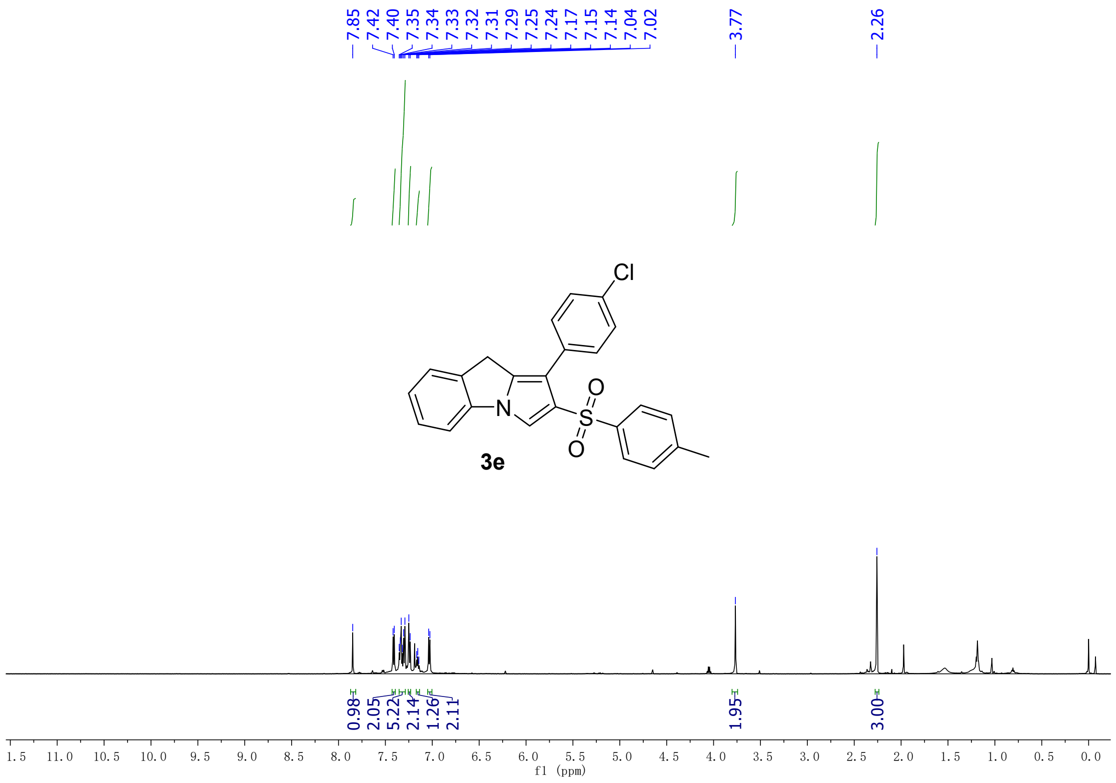

^{13}C NMR of **3e** in $CDCl_3$ (151 MHz, $CDCl_3$)

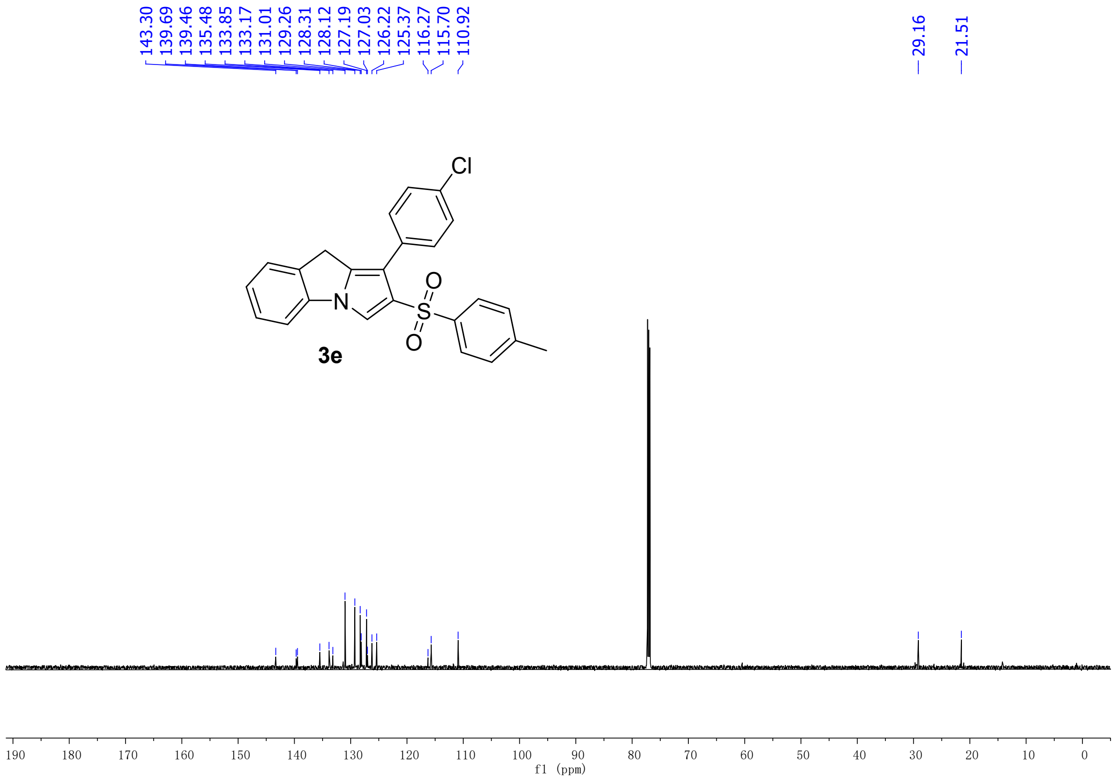

^{1}H NMR of **3f** in $CDCl_3$ (600 MHz, $CDCl_3$)

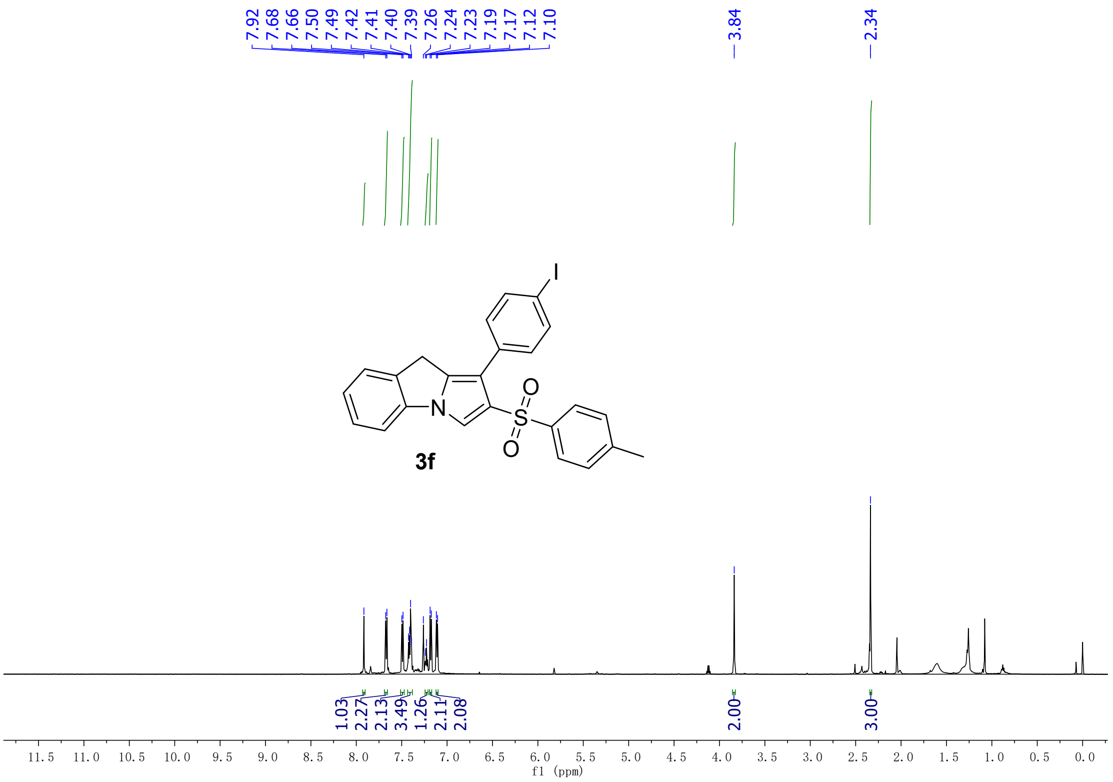

^{13}C NMR of **3f** in $CDCl_3$ (151 MHz, $CDCl_3$)

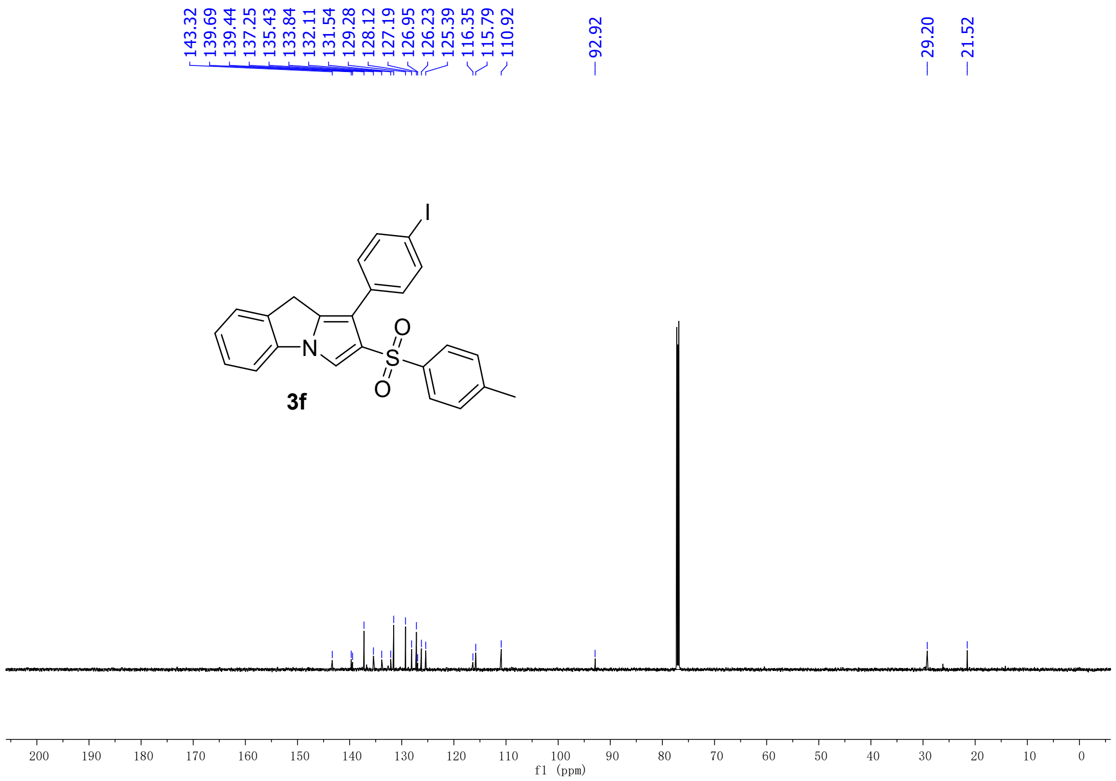

^{1}H NMR of **3g** in $CDCl_3$ (600 MHz, $CDCl_3$)

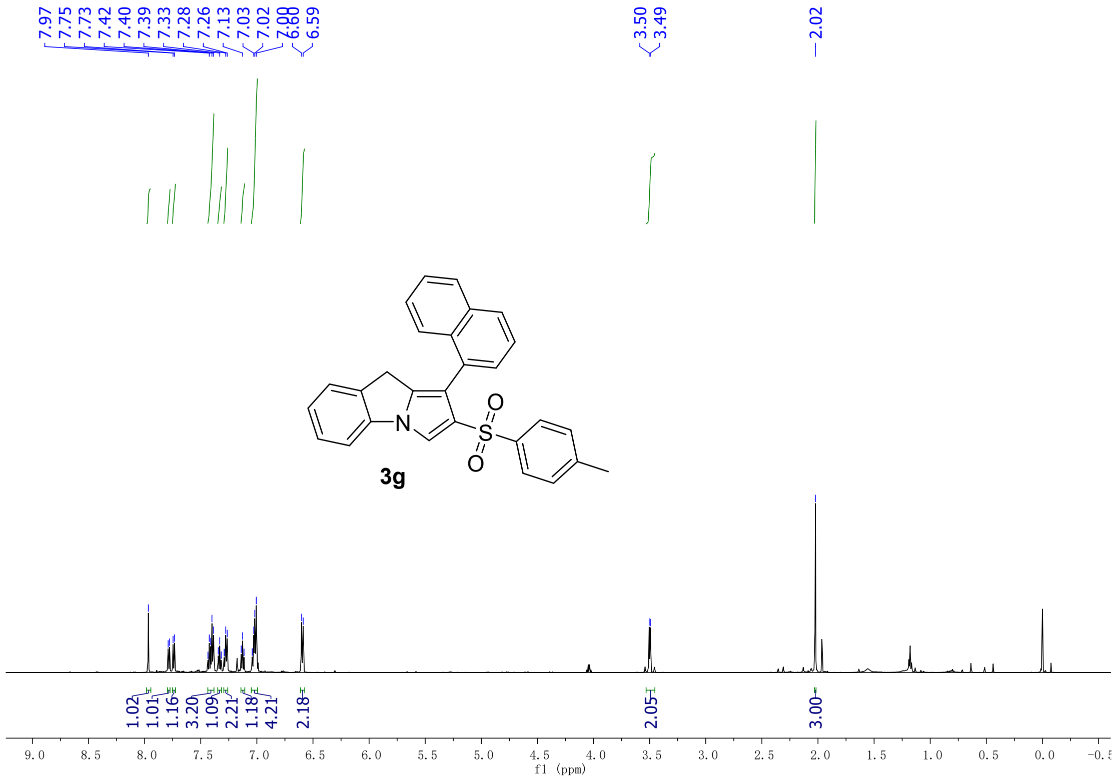

^{13}C NMR of **3g** in $CDCl_3$ (151 MHz, $CDCl_3$)

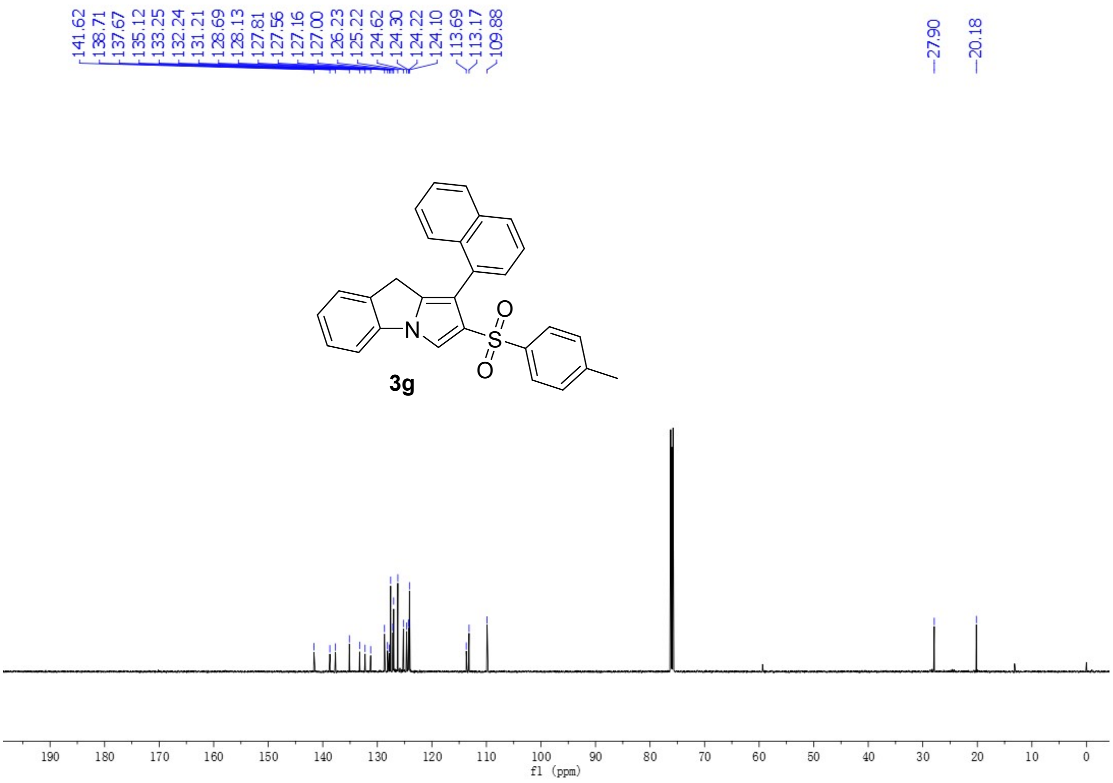

^{1}H NMR of **3h** in $CDCl_3$ (600 MHz, $CDCl_3$)

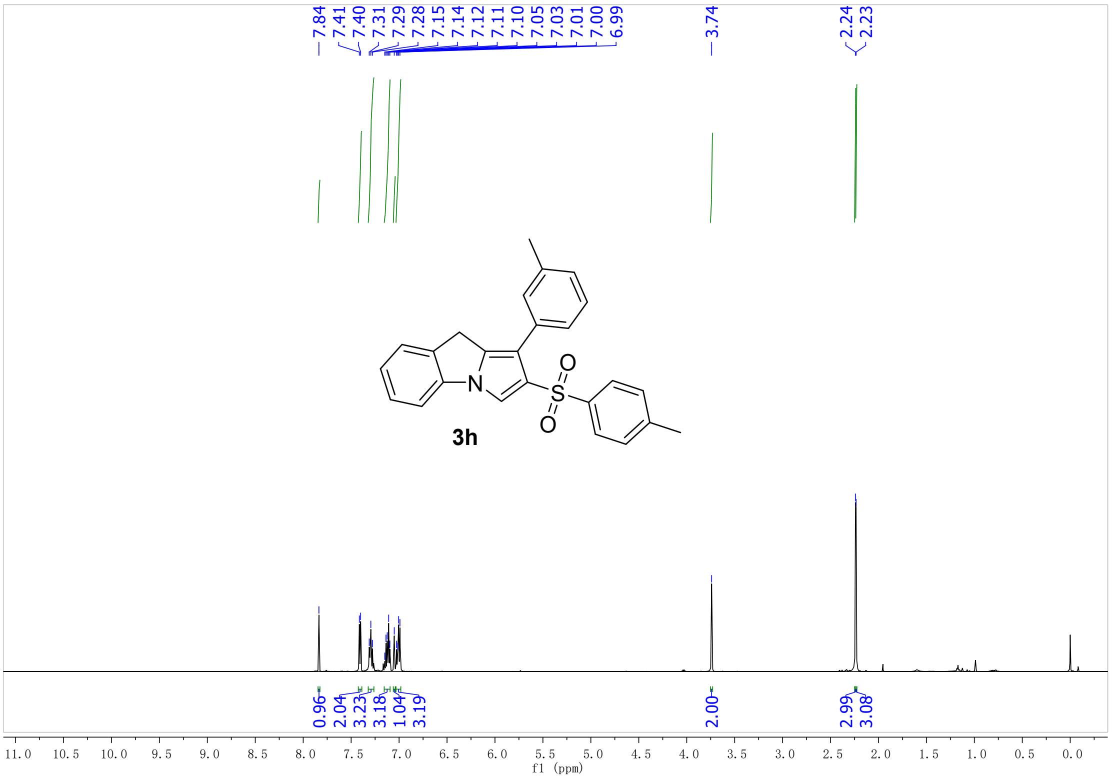

^{13}C NMR of **3h** in $CDCl_3$ (151 MHz, $CDCl_3$)

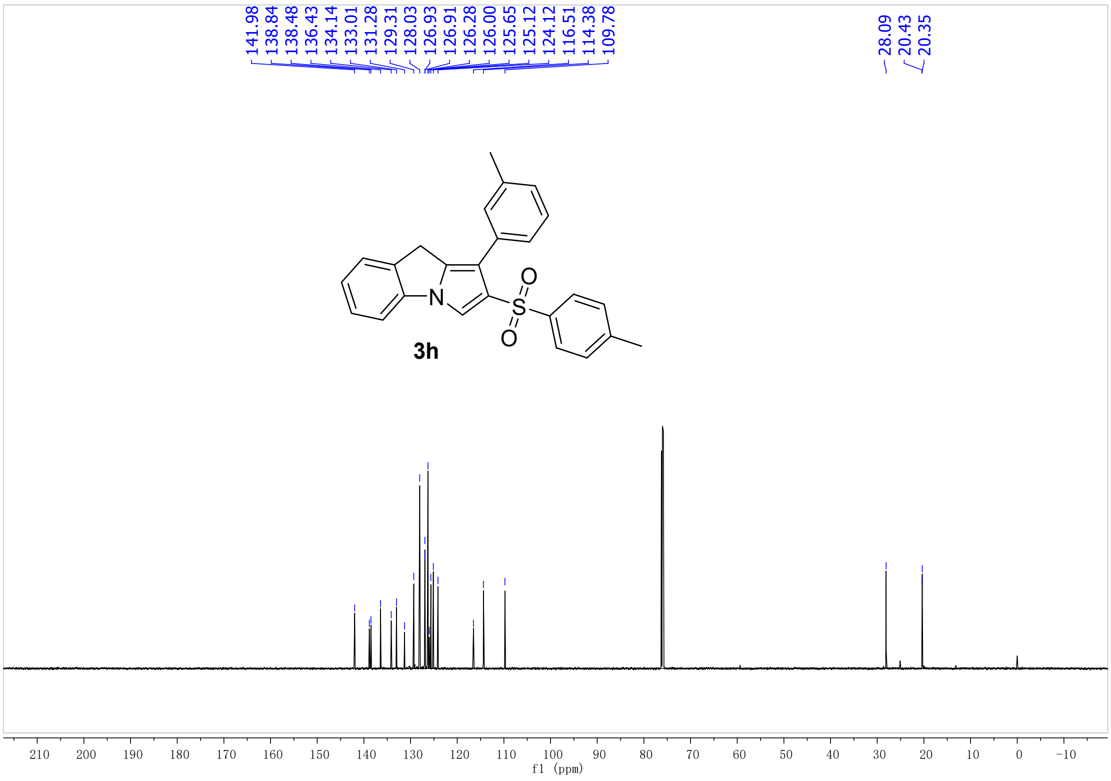

^{1}H NMR of **3i** in $CDCl_3$ (600 MHz, $CDCl_3$)

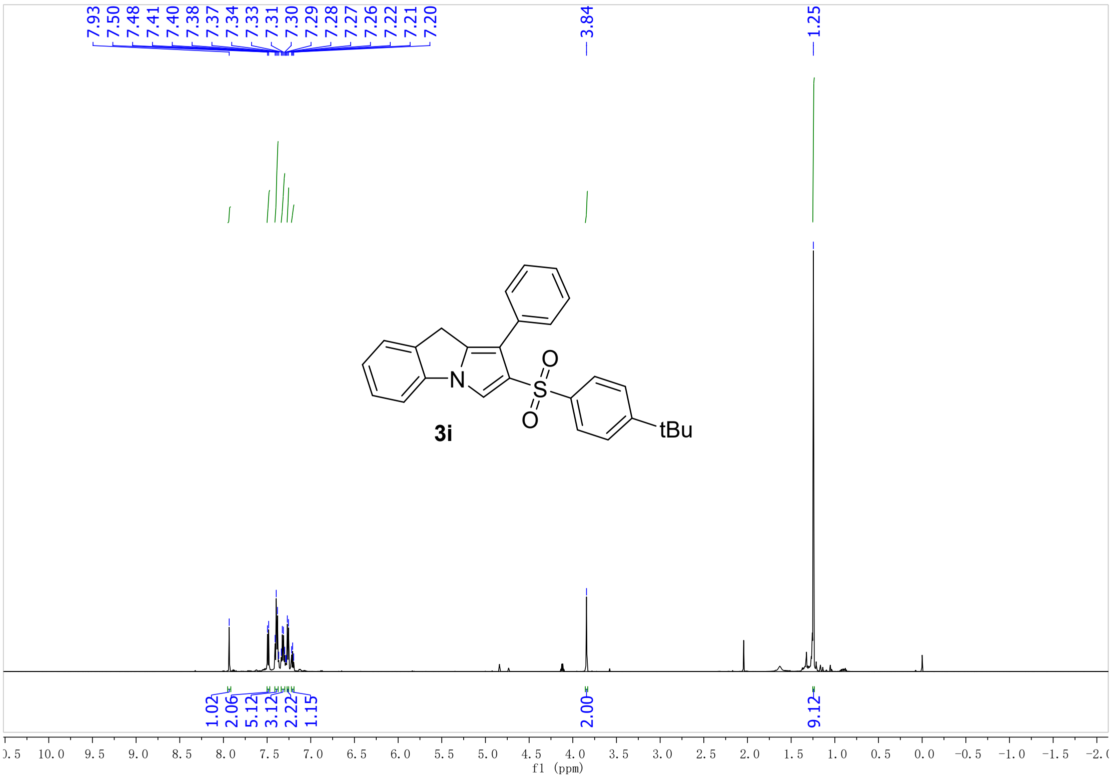

^{13}C NMR of **3i** in $CDCl_3$ (151 MHz, $CDCl_3$)

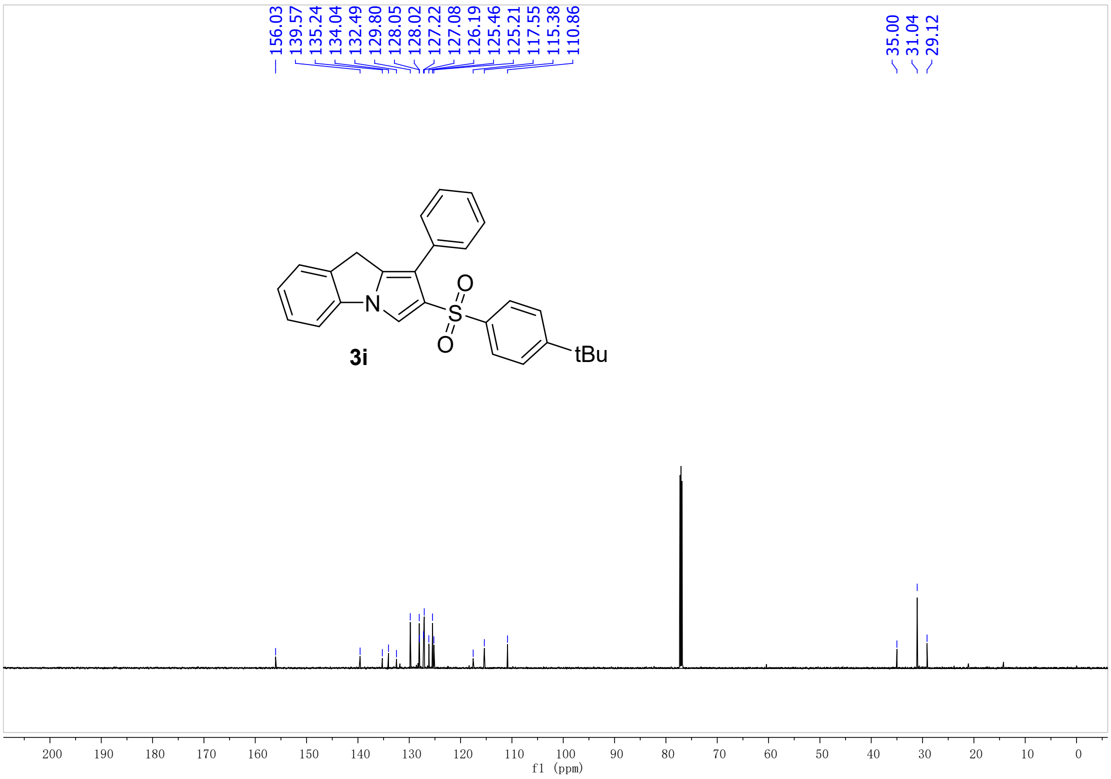

^{1}H NMR of **3j** in $CDCl_3$ (600 MHz, $CDCl_3$)

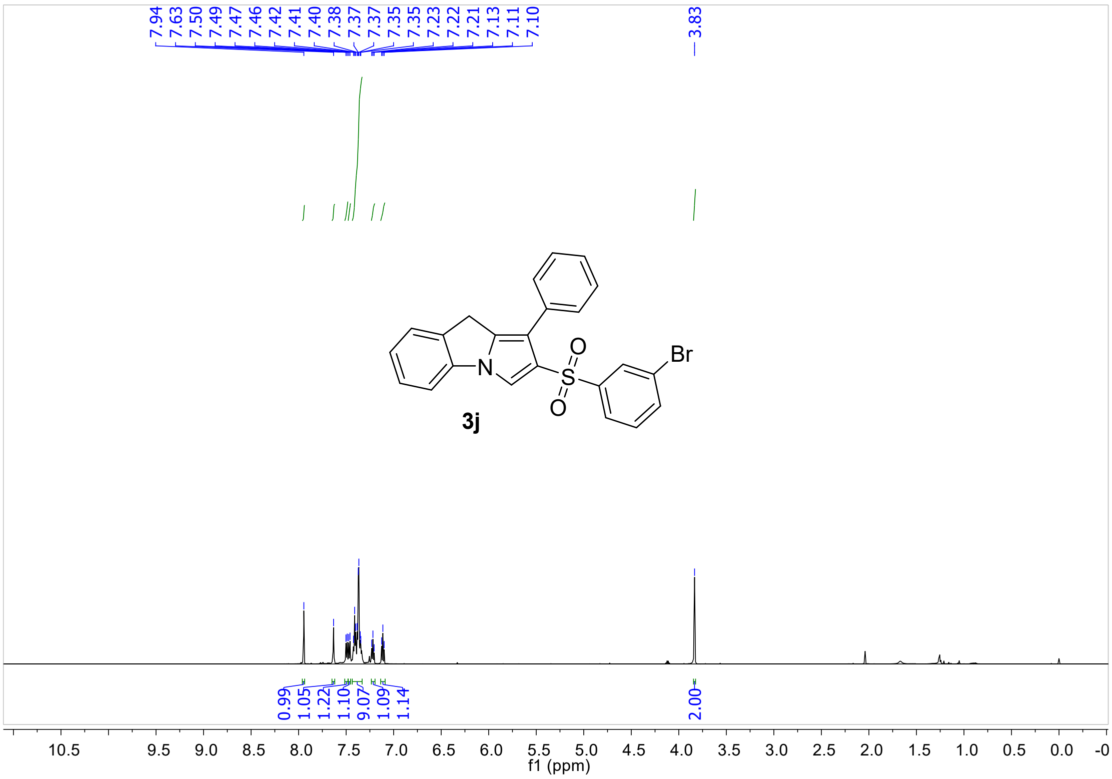

^{13}C NMR of **3j** in $CDCl_3$ (151 MHz, $CDCl_3$)

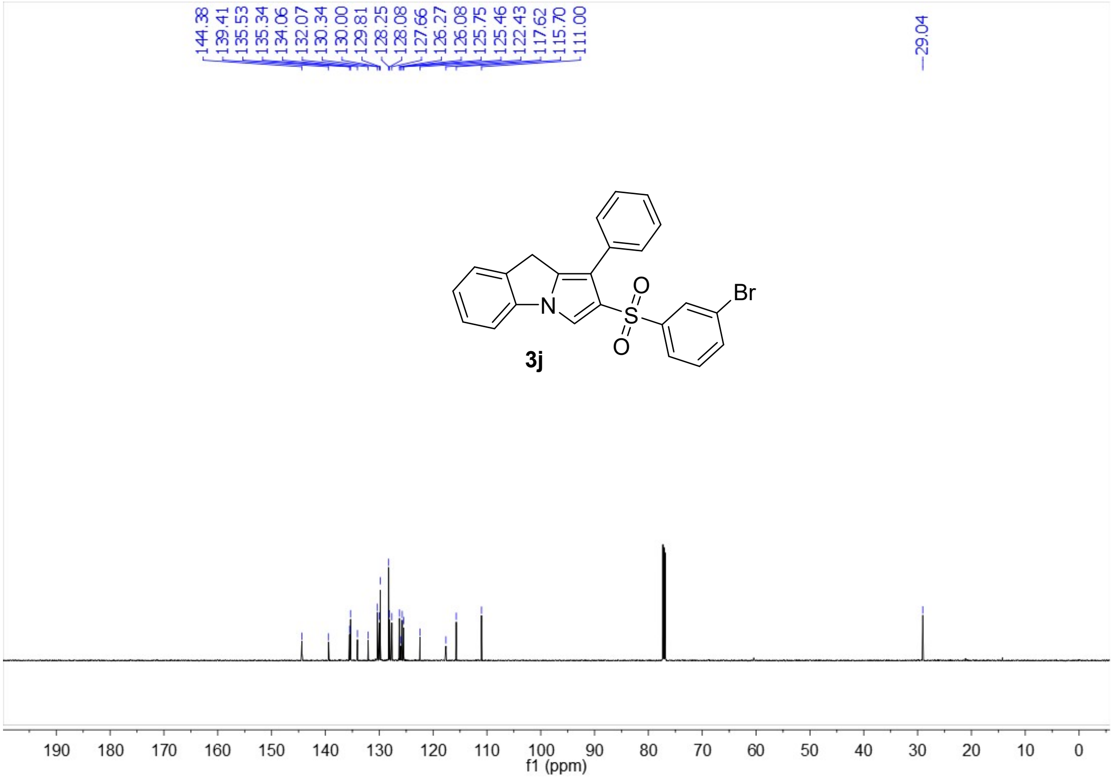

^{1}H NMR of **3k** in $CDCl_3$ (600 MHz, $CDCl_3$)

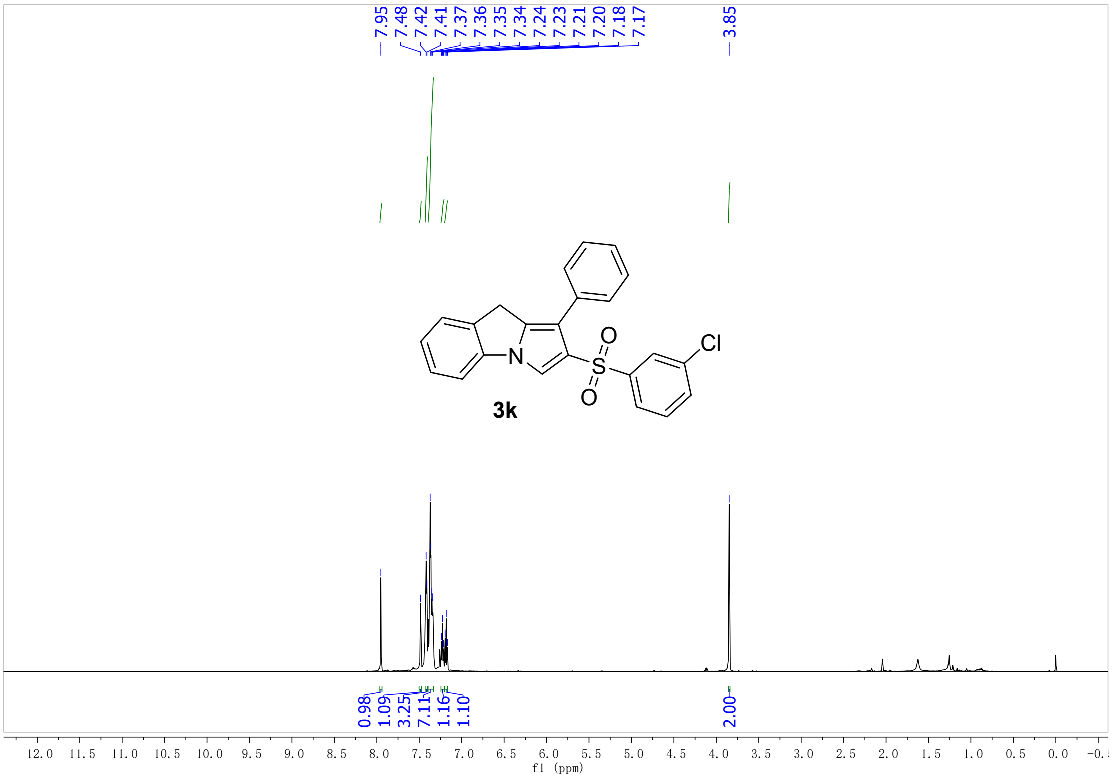

^{13}C NMR of **3k** in $CDCl_3$ (151 MHz, $CDCl_3$)

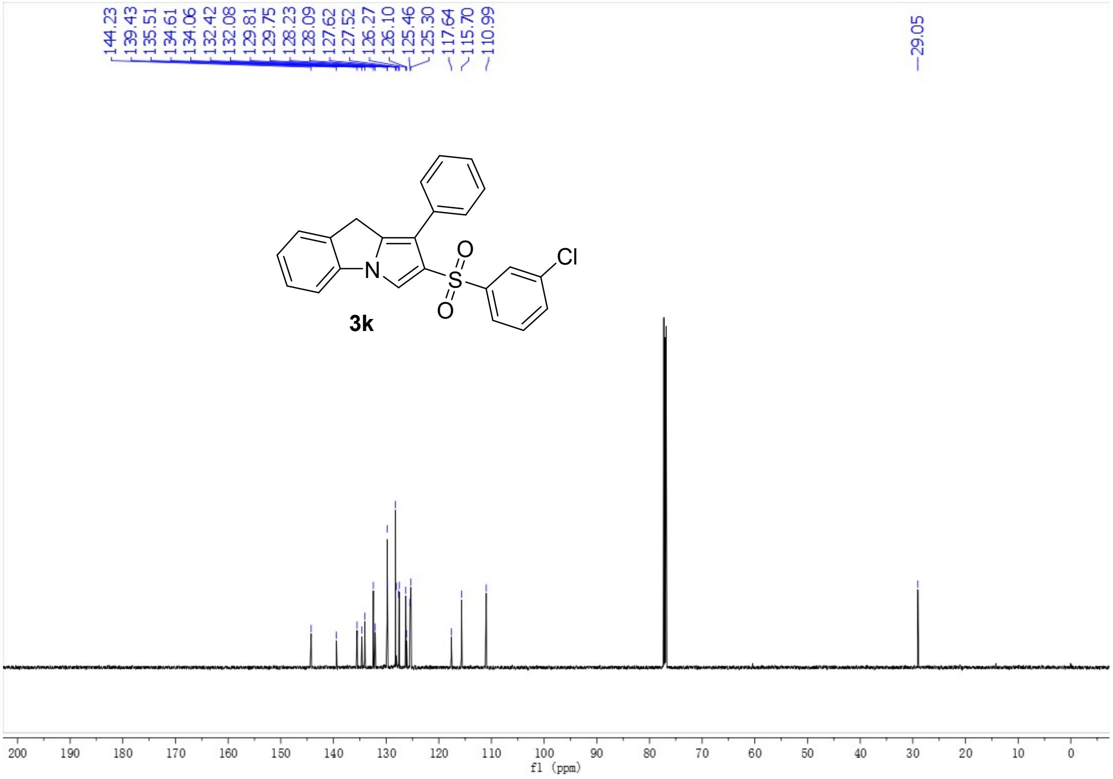

^{1}H NMR of **3l** in $CDCl_3$ (600 MHz, $CDCl_3$)

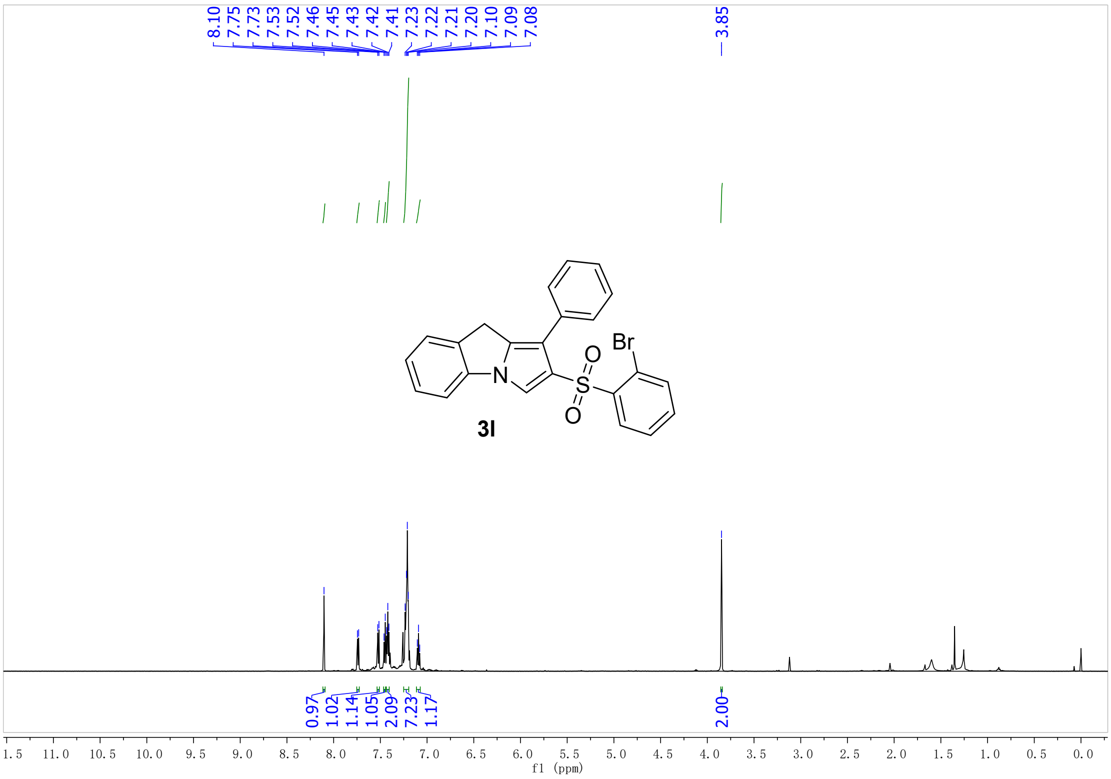

^{13}C NMR of **3l** in $CDCl_3$ (151 MHz, $CDCl_3$)

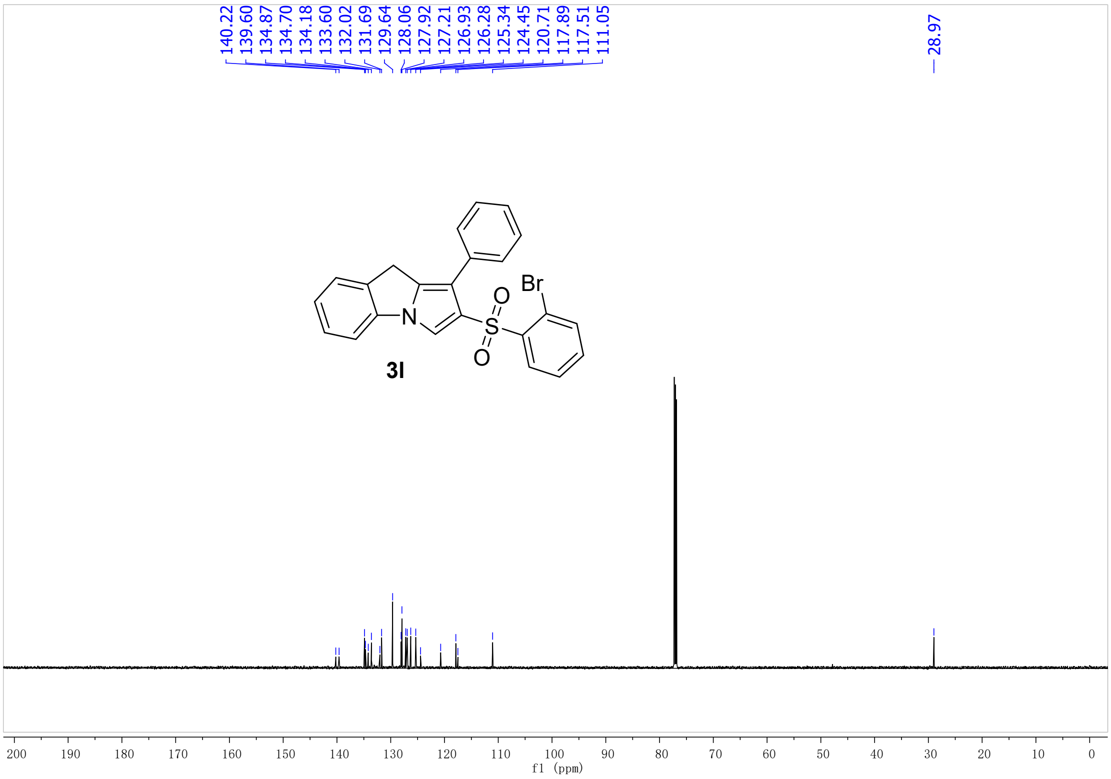

^{1}H NMR of **3m** in $CDCl_3$ (600 MHz, $CDCl_3$)

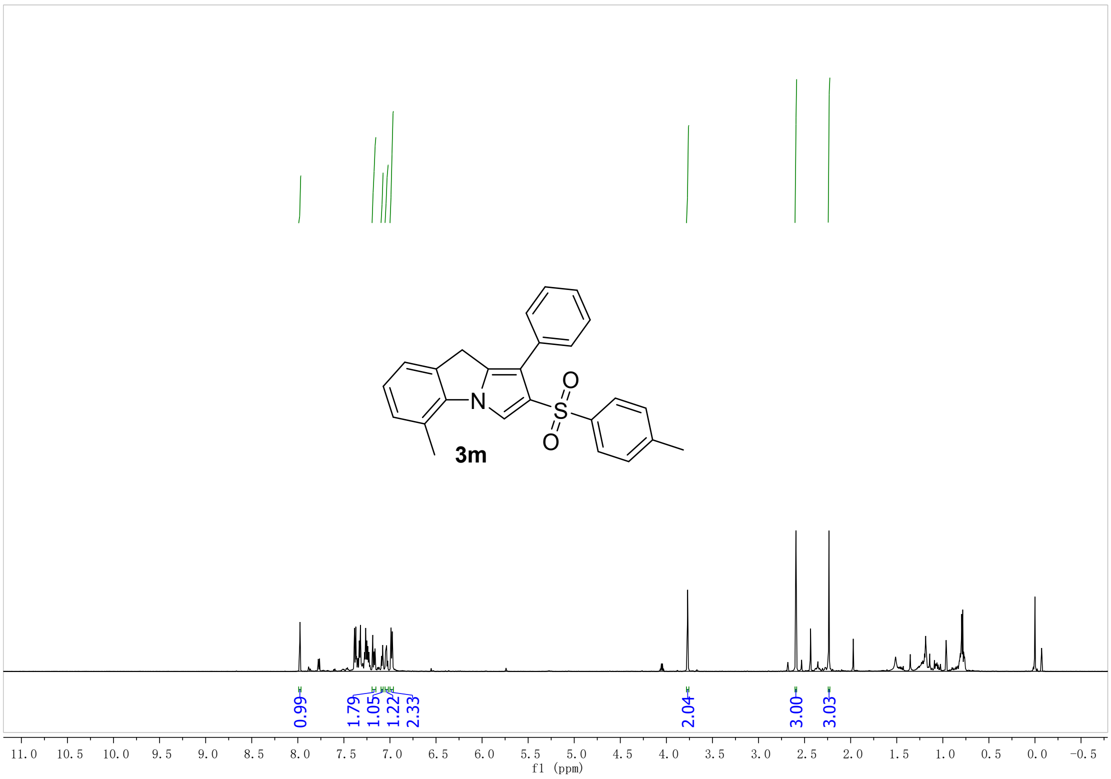

^{13}C NMR of **3m** in $CDCl_3$ (151 MHz, $CDCl_3$)

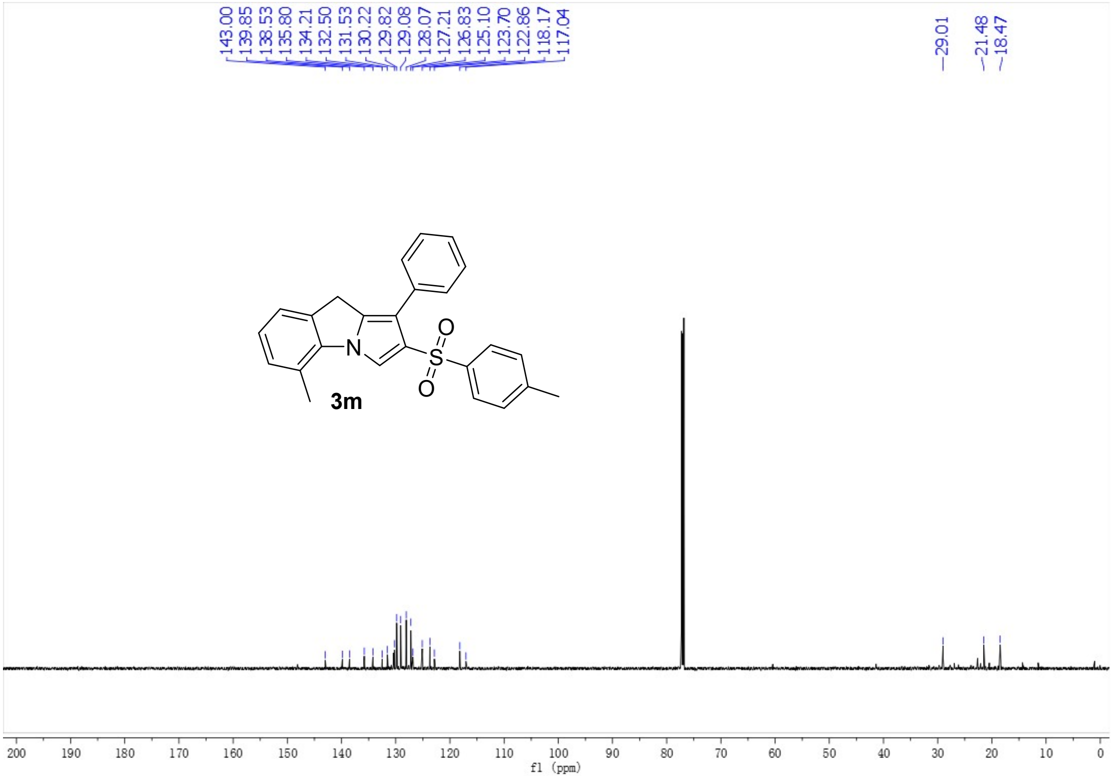

^{1}H NMR of **5a** in $CDCl_3$ (600 MHz, $CDCl_3$)

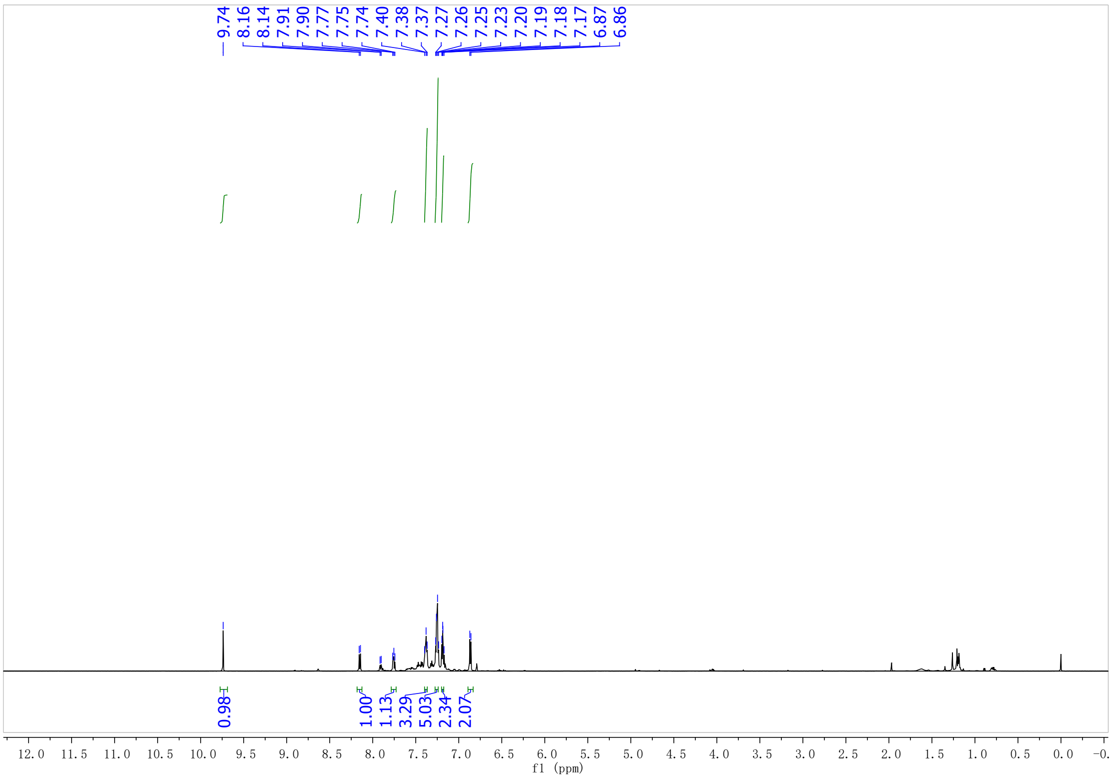

^{13}C NMR of **5a** in $CDCl_3$ (151 MHz, $CDCl_3$)

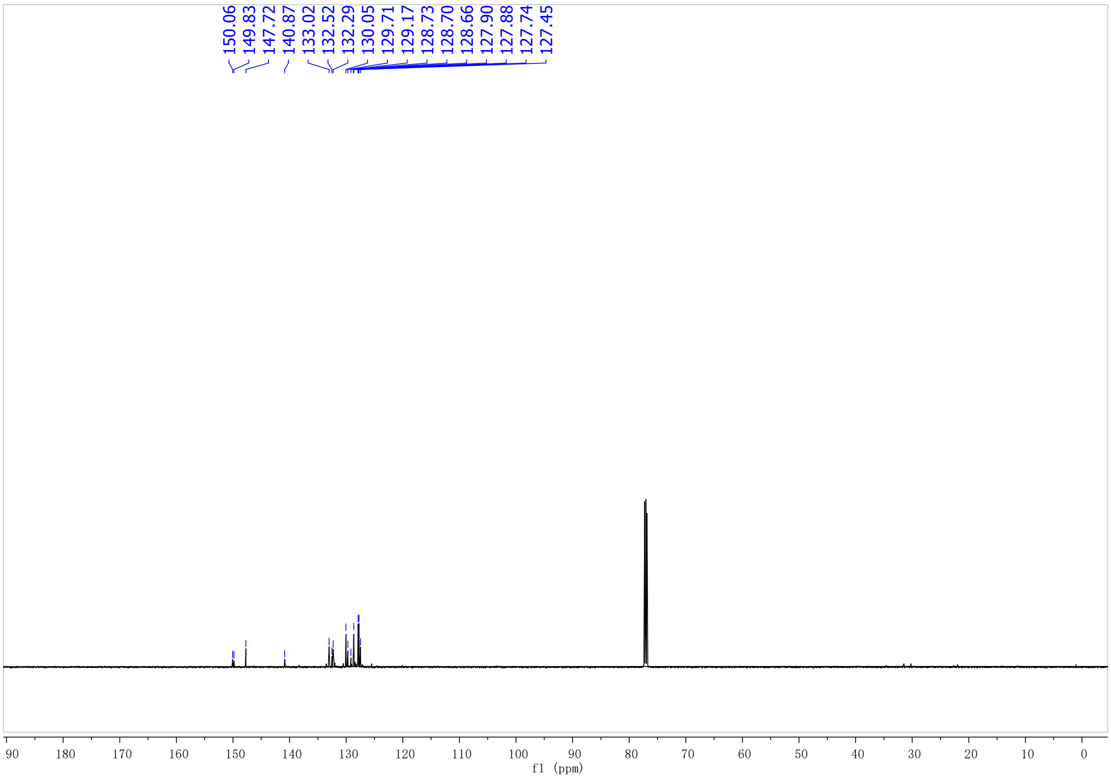

^{1}H NMR of **5b** in $CDCl_3$ (600 MHz, $CDCl_3$)

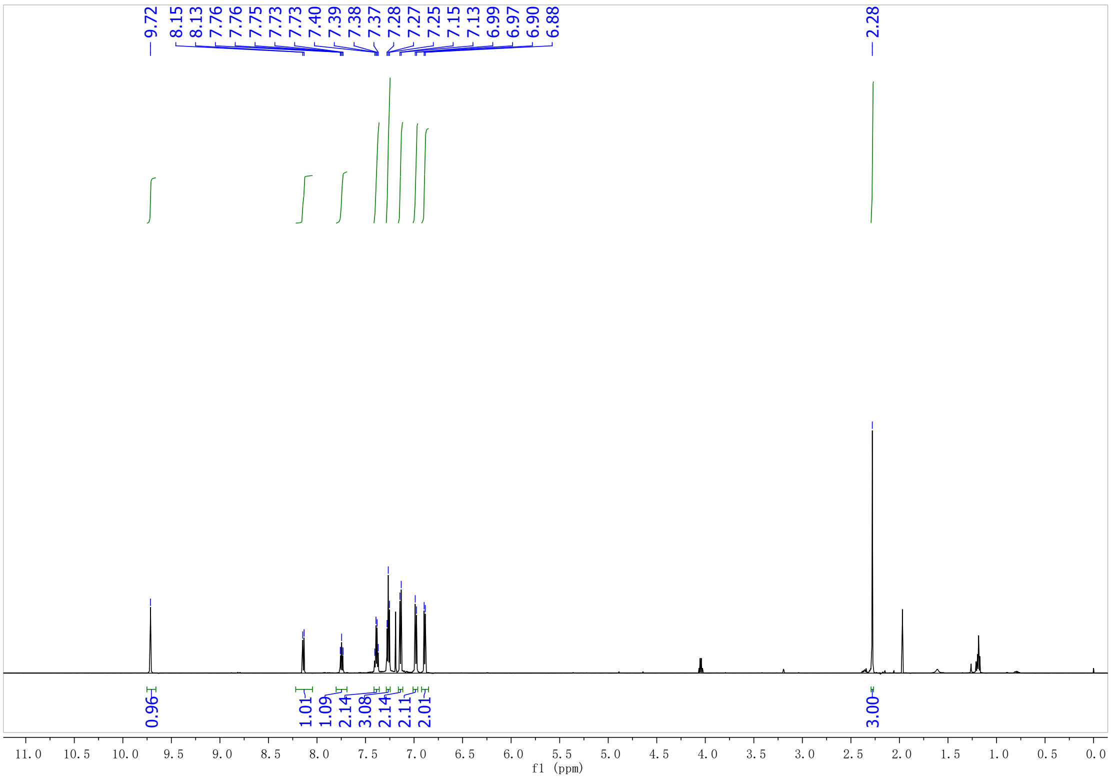

^{13}C NMR of **5b** in $CDCl_3$ (151 MHz, $CDCl_3$)

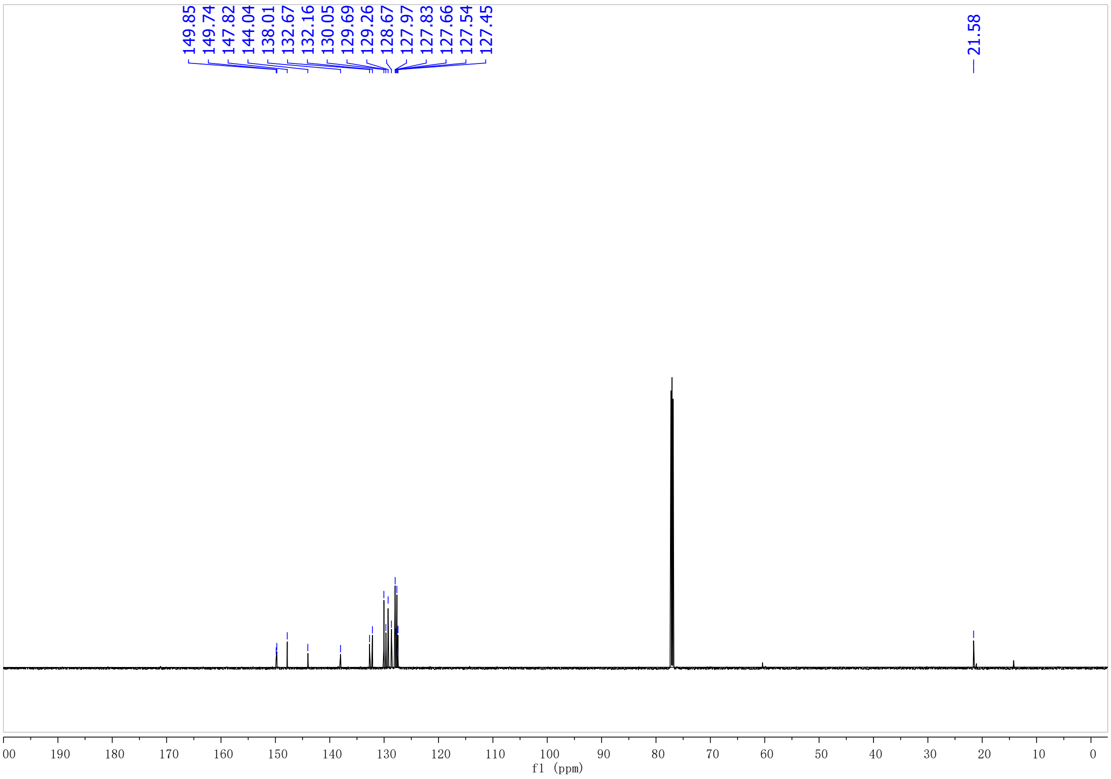

^{1}H NMR of **5c** in $CDCl_3$ (400 MHz, $CDCl_3$)

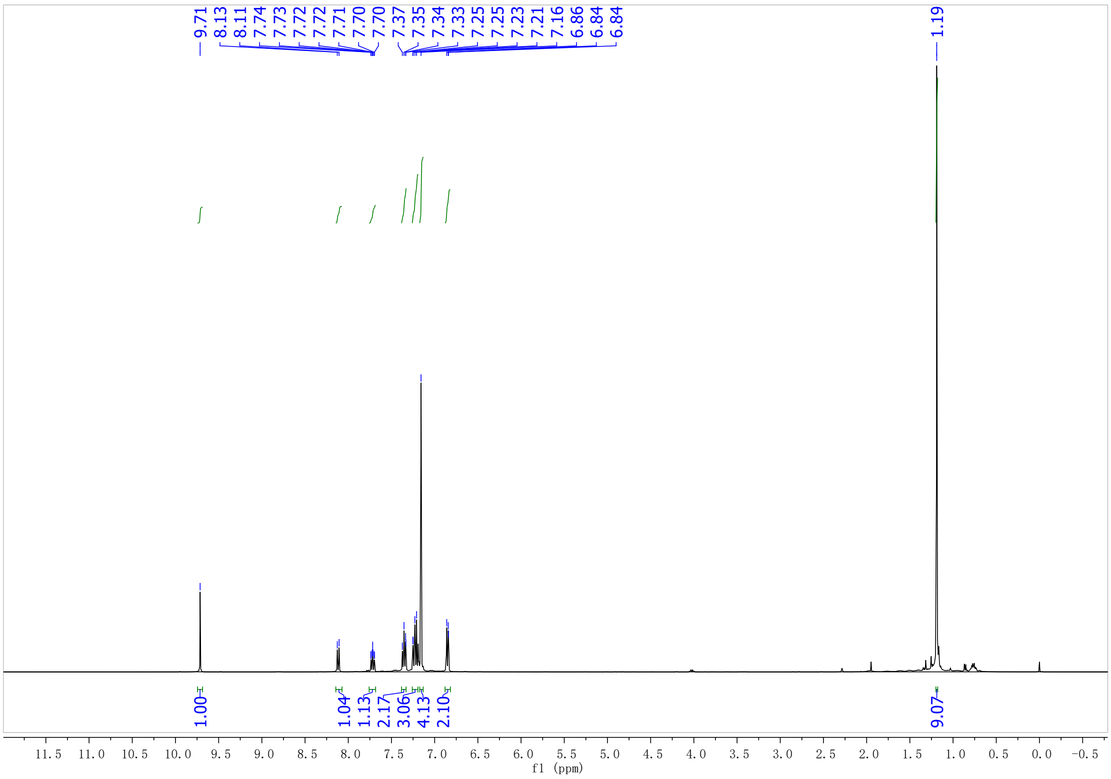

^{13}C NMR of **5c** in $CDCl_3$ (101 MHz, $CDCl_3$)

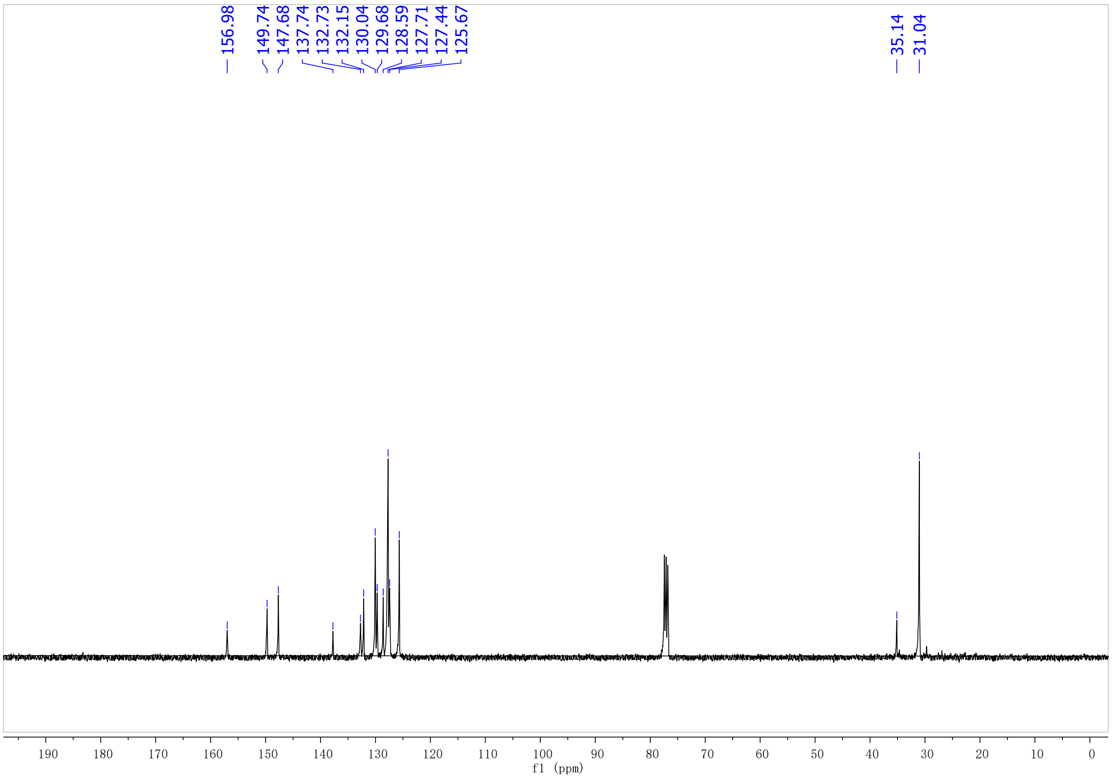

^{1}H NMR of **5d** in $CDCl_3$ (400 MHz, $CDCl_3$)

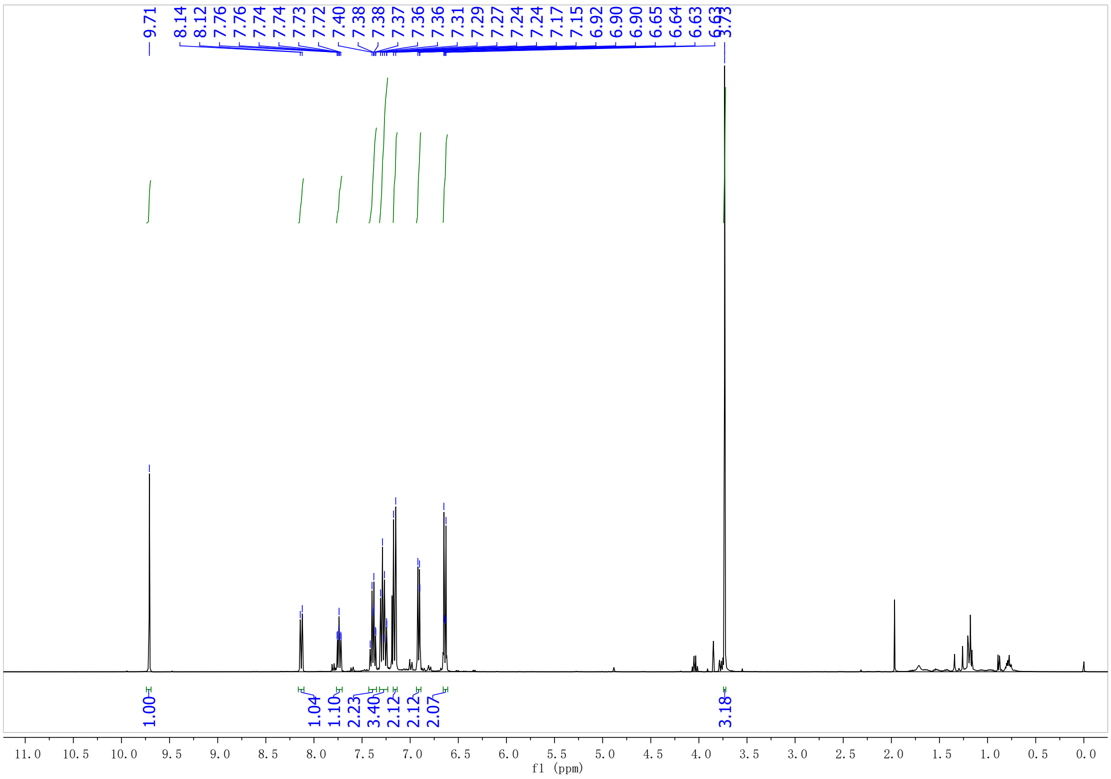

^{13}C NMR of **5d** in $CDCl_3$ (101 MHz, $CDCl_3$)

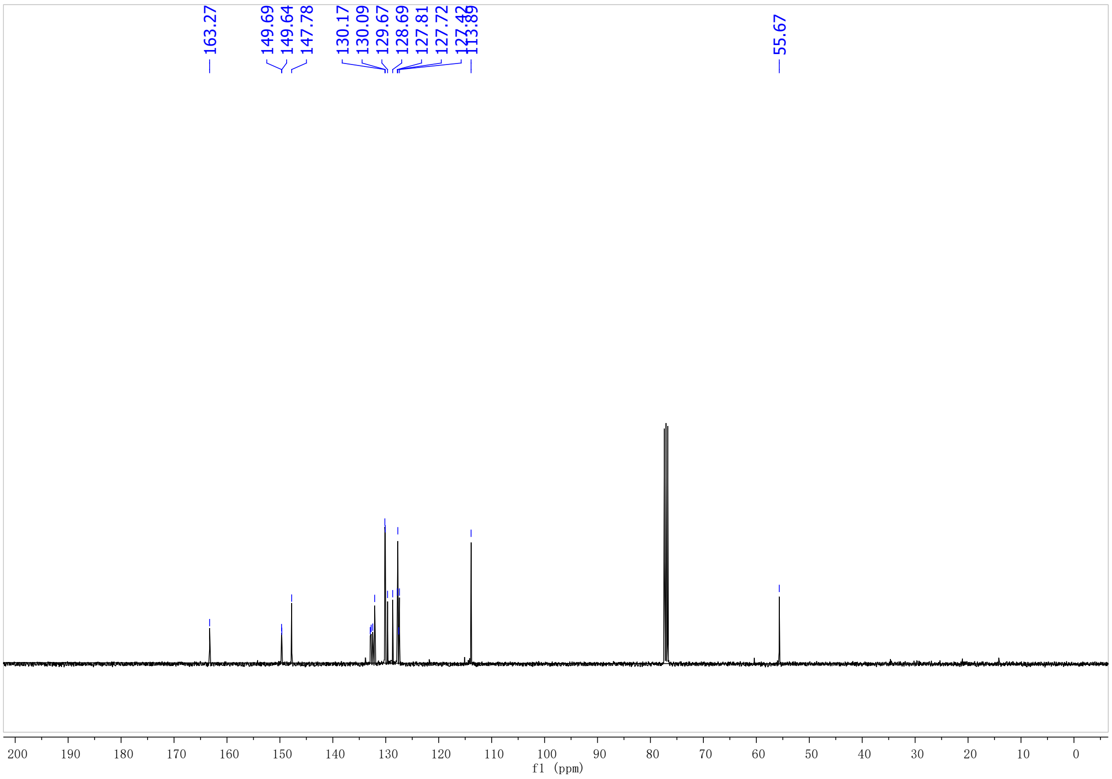

^{1}H NMR of **5e** in $CDCl_3$ (400 MHz, $CDCl_3$)

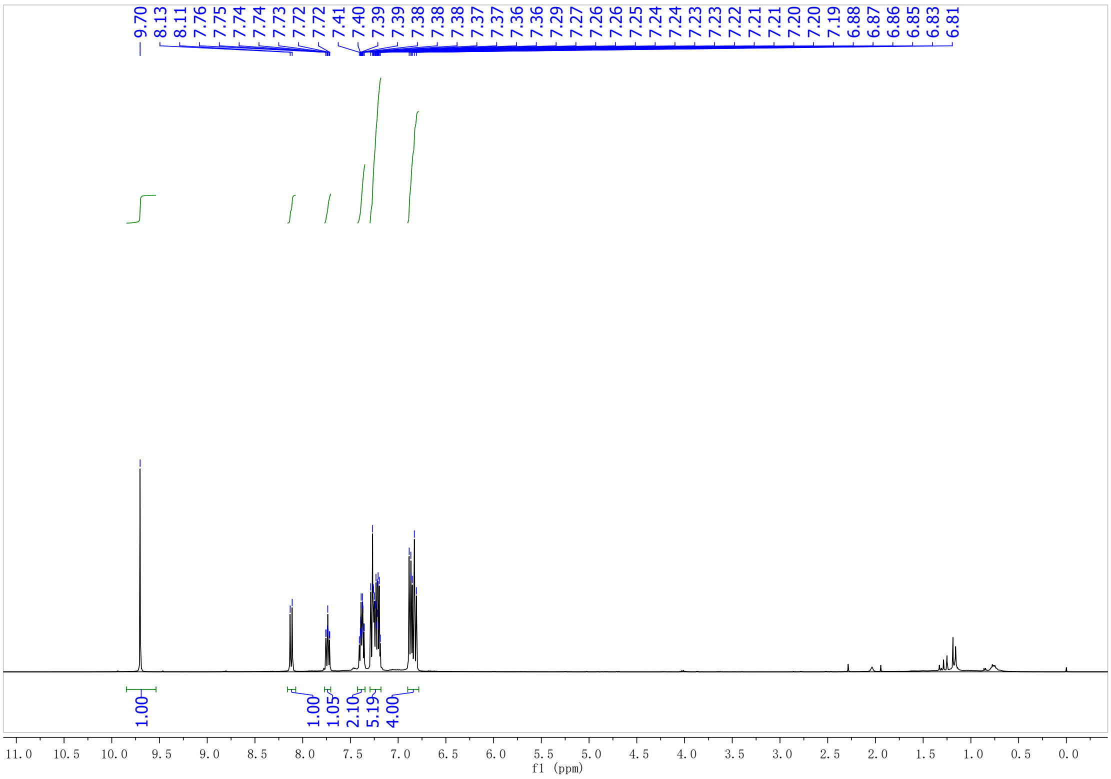

^{13}C NMR of **5e** in $CDCl_3$ (101 MHz, $CDCl_3$)

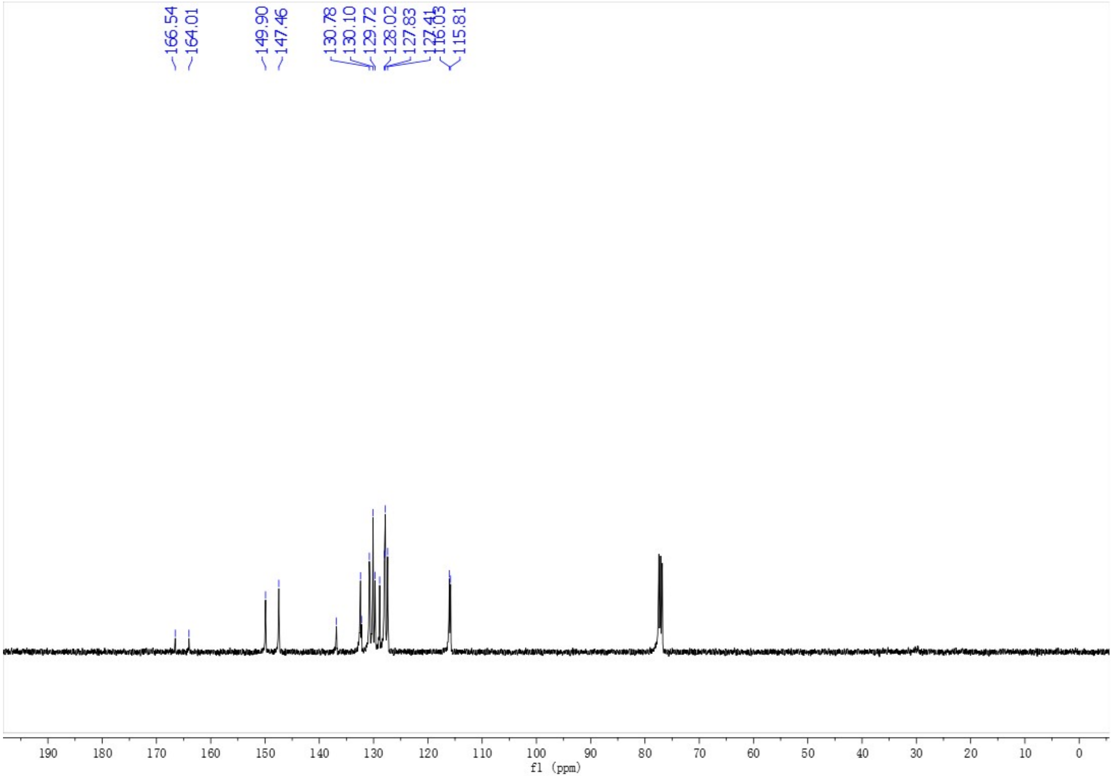

^{1}H NMR of **5f** in $CDCl_3$ (400 MHz, $CDCl_3$)

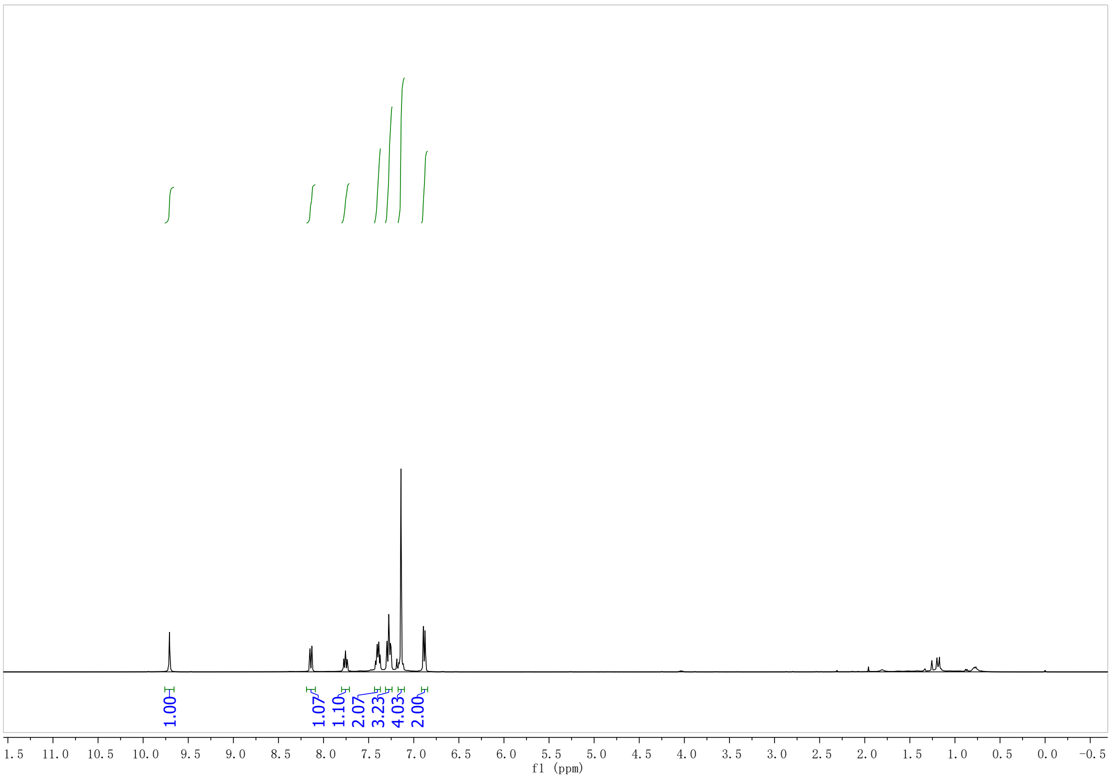

^{13}C NMR of **5f** in $CDCl_3$ (101 MHz, $CDCl_3$)

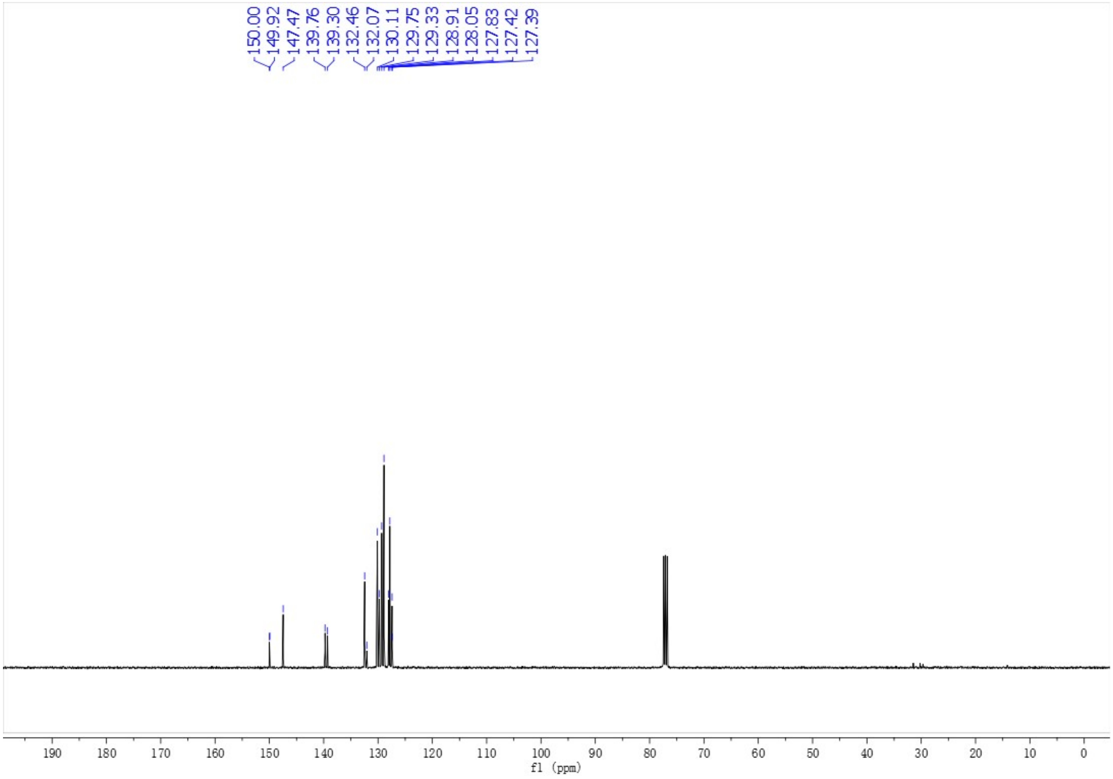

^{1}H NMR of **5g** in $CDCl_3$ (400 MHz, $CDCl_3$)

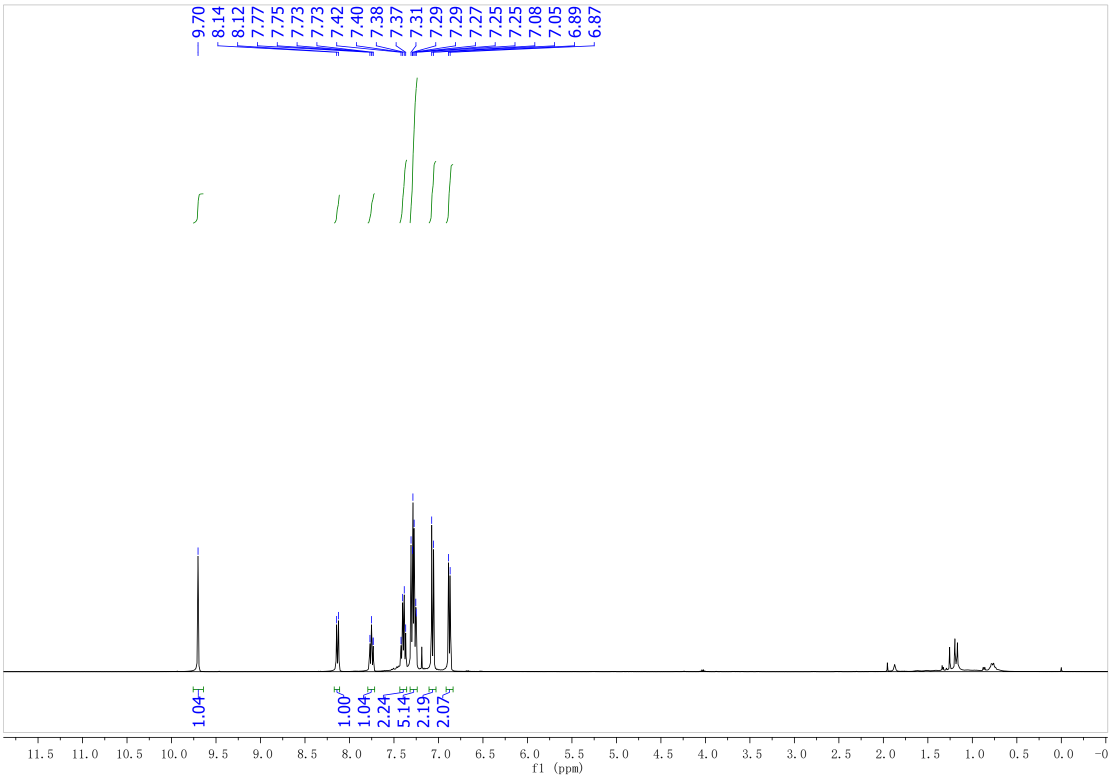

^{13}C NMR of **5g** in $CDCl_3$ (101 MHz, $CDCl_3$)

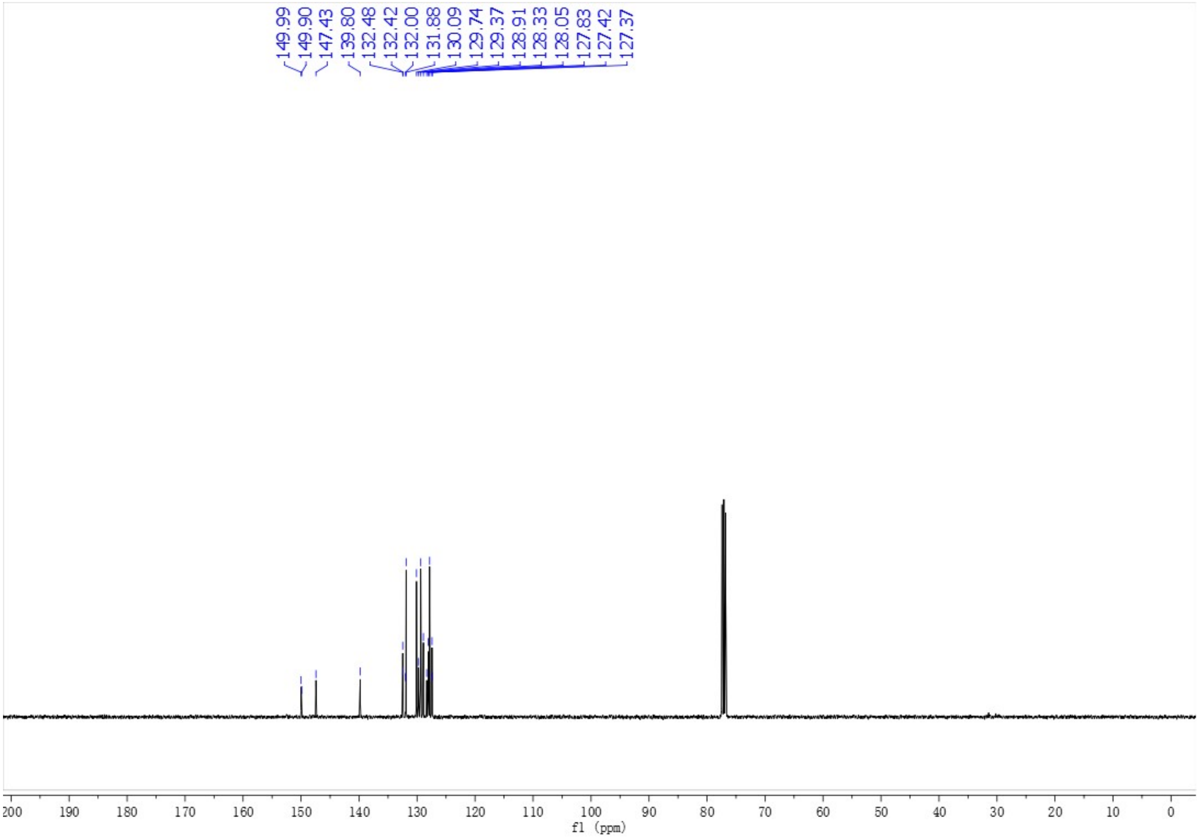

^{1}H NMR of **5h** in $CDCl_3$ (400 MHz, $CDCl_3$)

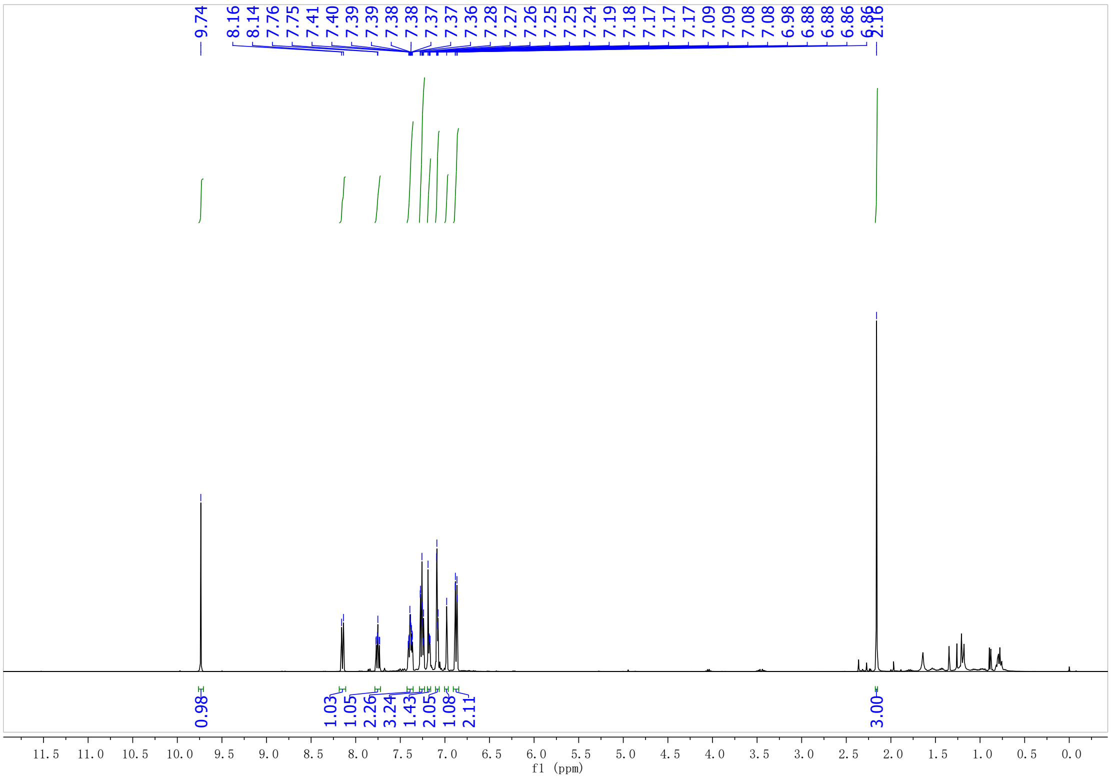

^{13}C NMR of **5h** in $CDCl_3$ (101 MHz, $CDCl_3$)

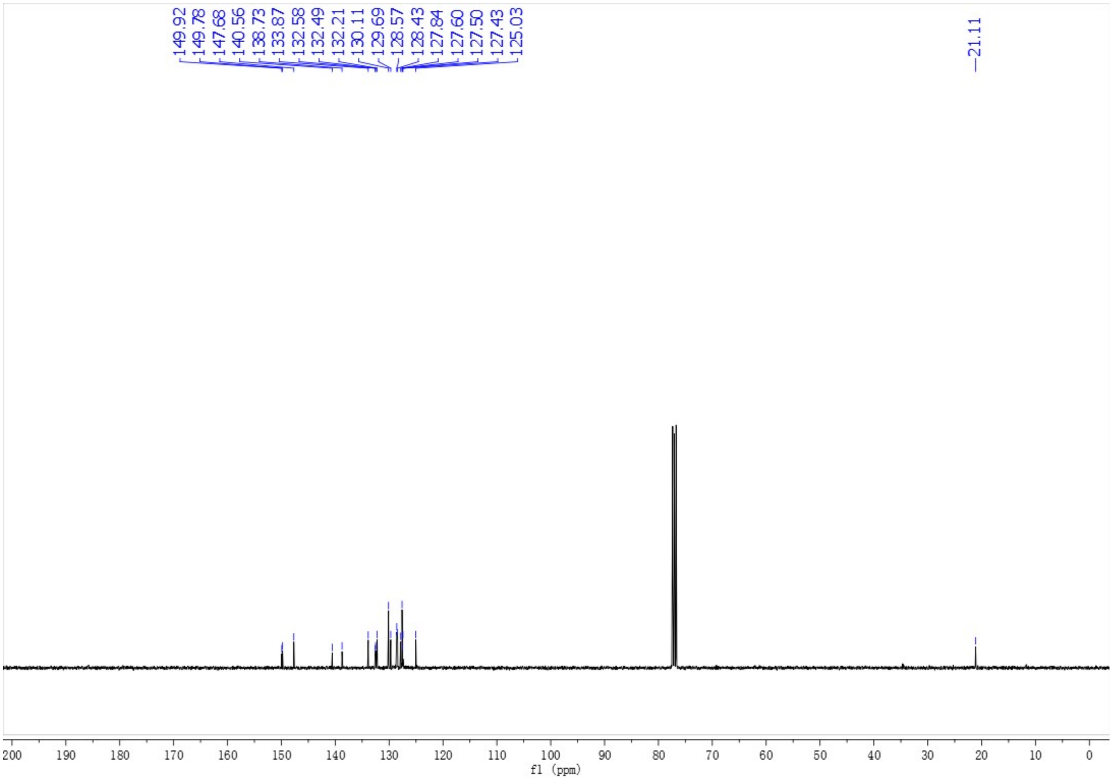

^{1}H NMR of **7** in $CDCl_3$ (600 MHz, $CDCl_3$)

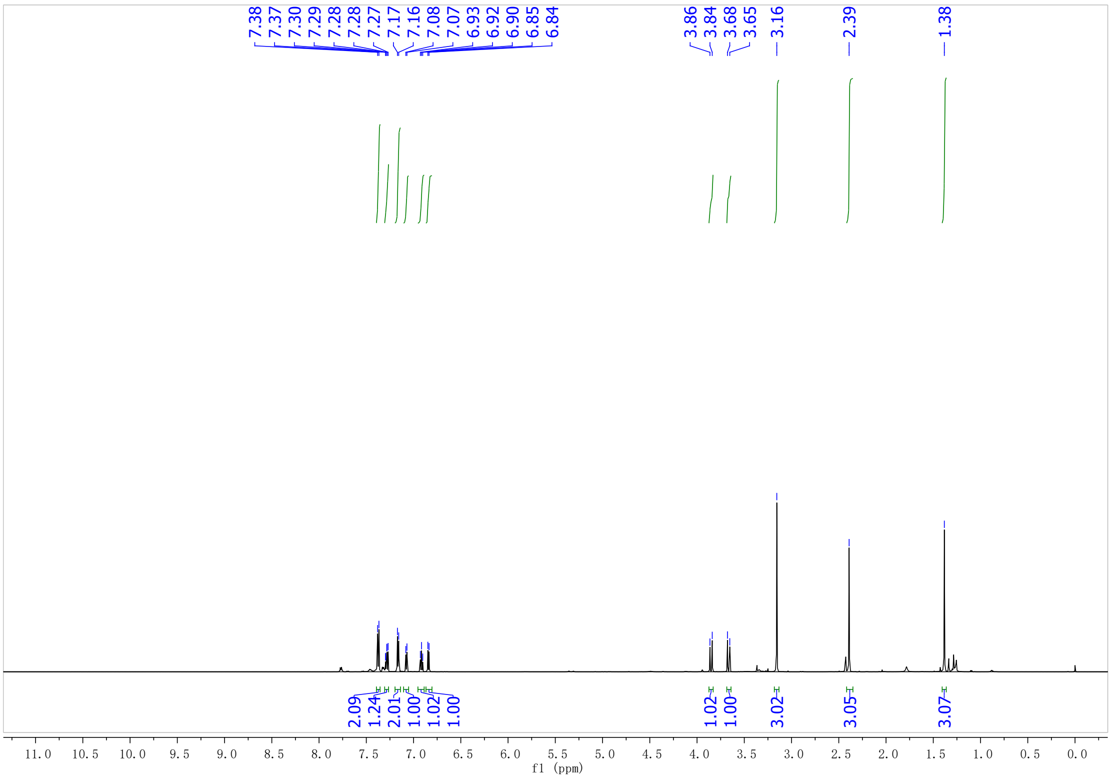
7.38
7.37
7.30
7.29
7.28
7.28
7.27
7.17
7.16
7.08
7.07
6.93
6.92
6.90
6.85
6.84
3.86
3.84
3.68
3.65
3.16
2.39
1.38
2.09
1.24
2.01
1.00
1.02
1.00
1.02
1.00
3.02
3.05
3.07
11.0
10.5
10.0
9.5
9.0
8.5
8.0
7.5
7.0
6.5
6.0
5.5
5.0
4.5
4.0
3.5
3.0
2.5
2.0
1.5
1.0
0.5
0.0
f1 (ppm)
